# Association of Genetic Liability to Psychotic Experiences With Neuropsychotic Disorders and Traits

**DOI:** 10.1001/jamapsychiatry.2019.2508

**Published:** 2019-09-25

**Authors:** Sophie E. Legge, Hannah J. Jones, Kimberley M. Kendall, Antonio F. Pardiñas, Georgina Menzies, Matthew Bracher-Smith, Valentina Escott-Price, Elliott Rees, Katrina A. S. Davis, Matthew Hotopf, Jeanne E. Savage, Danielle Posthuma, Peter Holmans, George Kirov, Michael J. Owen, Michael C. O’Donovan, Stanley Zammit, James T. R. Walters

**Affiliations:** 1 MRC Centre for Neuropsychiatric Genetics and Genomics, Division of Psychological Medicine and Clinical Neurosciences, School of Medicine, Cardiff University, Cardiff, United Kingdom; 2 Centre for Academic Mental Health, Department of Population Health Sciences, Bristol Medical School, University of Bristol, Bristol, United Kingdom; 3 Medical Research Centre, Integrative Epidemiology Unit, University of Bristol, Bristol, United Kingdom; 4 National Institute for Health Research Bristol Biomedical Research Centre, University Hospitals Bristol National Health Service Foundation Trust, University of Bristol, Bristol, United Kingdom; 5 UK-Dementia Research Institute at Cardiff, Division of Psychological Medicine and Clinical Neurosciences, School of Medicine, Cardiff University, Cardiff, United Kingdom; 6 Institute of Psychiatry, Psychology & Neuroscience, King’s College London, London, United Kingdom; 7 South London and Maudsley National Health Service Foundation Trust, London, United Kingdom; 8 Department of Complex Trait Genetics, Centre for Neurogenomics and Cognitive Research, Amsterdam Neuroscience, Vrije Universiteit, Amsterdam, the Netherlands

## Abstract

**Importance:**

Psychotic experiences, such as hallucinations and delusions, are reported by approximately 5% to 10% of the general population, although only a small proportion develop psychotic disorders such as schizophrenia. Studying the genetic causes of psychotic experiences in the general population, and its association with the genetic causes of other disorders, may increase the understanding of their pathologic significance.

**Objectives:**

To determine whether genetic liability to psychotic experiences is shared with schizophrenia and/or other neuropsychiatric disorders and traits and to identify genetic loci associated with psychotic experiences.

**Design, Setting and Participants:**

Analyses of genetic correlation, polygenic risk scores, and copy number variation were performed using data from participants in the UK Biobank from April 1, 2018, to March 20, 2019, to assess whether genetic liability to psychotic experiences is shared with schizophrenia and/or other neuropsychiatric disorders and traits. Genome-wide association studies of psychotic experience phenotypes were conducted to identify novel genetic loci. Participants in the final analyses after exclusions included 6123 individuals reporting any psychotic experience, 2143 individuals reporting distressing psychotic experiences, and 3337 individuals reporting multiple occurrences of psychotic experiences. A total of 121 843 individuals who did not report a psychotic experience formed the comparator group. Individuals with a psychotic disorder were excluded from all analyses.

**Main Outcomes and Measures:**

Genetic associations with psychotic experience phenotypes.

**Results:**

The study included a total of 127 966 participants (56.0% women and 44.0% men; mean [SD] age, 64.0 [7.6] years). Psychotic experiences were genetically correlated with major depressive disorder, schizophrenia, autism spectrum disorder, and attention-deficit/hyperactivity disorder. Analyses of polygenic risk scores identified associations between psychotic experiences and genetic liability for major depressive disorder, schizophrenia, bipolar disorder, autism spectrum disorder, and attention-deficit/hyperactivity disorder. Individuals reporting psychotic experiences had an increased burden of copy number variations previously associated with schizophrenia (odds ratio [OR], 2.04; 95% CI, 1.39-2.98; *P* = 2.49 × 10^−4^) and neurodevelopmental disorders more widely (OR, 1.75; 95% CI, 1.24-2.48; *P* = 1.41 × 10^−3^). Genome-wide association studies identified 4 significantly associated loci, including a locus in Ankyrin-3 (*ANK3* [GenBank NM_020987]) (OR, 1.16; 95% CI, 1.10-1.23; *P* = 3.06 × 10^−8^) with any psychotic experience, and a locus in cannabinoid receptor 2 gene (*CNR2* [GenBank NM_001841]) (OR, 0.66; 95% CI, 0.56-0.78; *P* = 3.78 × 10^−8^) with distressing psychotic experiences. The genome-wide association study of any psychotic experience had a low single-nucleotide polymorphism–based heritability estimate (*h*^2^ = 1.71%; 95% CI, 1.02%-2.40%).

**Conclusions and Relevance:**

A large genetic association study of psychotic experiences from the population-based UK Biobank sample found support for a shared genetic liability between psychotic experiences and schizophrenia, major depressive disorder, bipolar disorder, and neurodevelopmental disorders.

## Key Points

**Question:**

Is the genetic liability to psychotic experiences shared with schizophrenia and/or other neuropsychiatric disorders and traits?

**Findings:**

In this cohort study, genetic correlation, polygenic risk score, and copy number variation analyses indicated a shared genetic liability between psychotic experiences and major depressive disorder, schizophrenia, bipolar disorder, and neurodevelopmental disorders. Genome-wide association studies identified 4 genetic loci associated with psychotic experiences including loci in *ANK3* and *CNR2*.

**Meaning:**

Findings suggest that the genetic liability of psychotic experiences is shared with several psychiatric disorders, which include, but is not specific to, schizophrenia.

## Teaser

This cohort study uses data from the UK Biobank to examine whether genetic liability to psychotic experiences is shared with schizophrenia and/or other neuropsychiatric disorders and traits and identify genetic loci associated with psychotic experiences.

## Introduction

Psychotic experiences, such as hallucinations and delusions, are features of psychiatric disorders (eg, schizophrenia), but they are also reported by approximately 5% to 10% of the general population.^1,2^ Psychotic experiences are considered to be symptoms of psychiatric illness only if they co-occur with other features of that disorder, including some aspect of psychosocial impairment. It is currently unclear whether psychotic experiences in the general population are: (1) on a spectrum that, at the extreme, is associated specifically with schizophrenia, (2) largely unassociated with the psychotic symptoms experienced in schizophrenia and other major mental disorders, or (3) associated with liability to major mental disorders more generally.

Twin studies and genome-wide association studies (GWASs) have provided evidence that psychotic experiences are heritable (30%-50% from twin studies,^3,4,5^ and 3%-17% for single-nucleotide polymorphism [SNP]–heritability estimates^6,7^), indicating that common genetic variants play a role in their liability. There have been 3 GWASs of psychotic experiences to date in adolescent samples^7,8,9^ and no reported genome-wide significant findings. Although there was an initial assumption that psychotic experiences in adolescence would specifically increase the risk for schizophrenia in later life, epidemiologic evidence suggests a nonspecific increased risk for broader psychopathologic characteristics.^10^ However, to date, no study has found strong evidence for an association between genetic liabilities for schizophrenia or any other mental disorder and psychotic experiences.^7,8,11,12,13^

Although many individuals with a lifetime history of psychotic experiences have their first experience in adolescence, nearly one-quarter of first-onset psychotic experiences occur after 40 years of age.^14^ Our aims were to use data from the UK Biobank to identify genetic loci associated with psychotic experiences reported by adults in a population-based study, and to determine whether genetic liability to psychotic experiences is shared with schizophrenia and/or other neuropsychiatric disorders and traits.

## Methods

### Sample

Study individuals were from the UK Biobank, a large prospective population-based cohort study of approximately 500 000 individuals between 40 and 69 years of age who were recruited from across the UK between 2006 and 2010.^15^ The North West Multi-Centre Ethics Committee granted ethical approval to UK Biobank and all participants provided written informed consent. This study was conducted under UK Biobank project numbers 13310 and 14421.

### Psychotic Experiences Phenotypes

A Mental Health Questionnaire (MHQ) was sent to all participants who provided an email address from July 13, 2016, to July 27, 2017, and was completed by 157 387 individuals (46.4% of those emailed; 31.4% of the total UK Biobank sample). For psychotic experiences, participants were asked about previous experience of visual hallucinations, auditory hallucinations, delusions of reference, delusions of persecution, as well as how often these experiences occurred and how distressing they found them (eAppendix in the Supplement). Individuals with a diagnosis of schizophrenia, bipolar disorder, or any other psychotic disorder were identified using all available sources (hospital records, death records, or self-report at the interview or from the MHQ) and were excluded from all analyses (full details in eMethods 1 in the Supplement).

We selected 3 primary phenotypes for GWASs (eFigure 1 in the Supplement): (1) any psychotic experience defined as a positive response to any of the 4 symptom questions (UK Biobank field IDs: 20463, 20468, 20471, and 20474); (2) a distressing psychotic experience, defined as any psychotic experience that was rated as “a bit,” “quite,” or “very” distressing (UK Biobank field ID: 20462); and (3) multiple occurrences of psychotic experiences, defined as any psychotic experience that occurred on more than 1 occasion (UK Biobank field IDs: 20465, 20470, 20473, and 20476). As a comparator group, we included individuals who provided a negative response to all 4 psychotic experience symptom questions. In addition, we investigated each individual psychotic experience symptom for association with polygenic risk scores (PRSs).

### Genetic Data

Genetic data for the study participants were provided by UK Biobank and the imputation and quality control procedures are fully described elsewhere.^16^ The data release contained 488 377 participants assayed on either the UK Biobank Axiom or the UK BiLEVE Axiom purpose-built arrays at the Affymetrix Research Services Laboratory. Standard quality control procedures were applied prior to imputation using Haplotype Reference Consortium^17^ and UK10K haplotype reference^18^ panels. We applied additional quality control filters to select high-quality SNPs,^19^ minor allele frequency greater than 0.01, imputation score greater than 0.8, missingness less than 0.05, Hardy-Weinberg equilibrium *P* value greater than 1 × 10^−6^ and removed SNPs imputed by the UK10K haplotype reference^18^ data set in accordance with guidance from the UK Biobank (http://www.ukbiobank.ac.uk/2017/07/important-note-about-imputed-genetics-data/). One member from each related pair with a kinship coefficient greater than 0.15 was excluded from analyses, preferentially retaining individuals who had experienced a psychotic experience, and were otherwise removed at random.

Analyses were restricted to individuals with a self-reported British and Irish ethnicity (UK Biobank field ID: 21000) and principal components supplied by UK Biobank^16^ (UK Biobank field ID: 22009) were used to make additional exclusions and control for population structure (described in eMethods 2 and eFigures 2 and 3 in the Supplement).

### GWAS Analysis

To identify genetic risk variants for psychotic experiences, association analysis was performed in SNPTEST, version 2.5.4^20^ using bgen, version 1.2 imputed dosage data.^21^ More than 7.5 million SNPs were included in each GWAS. An additive logistic regression model was used including as covariates the genotyping array, the top 5 principal components (as recommended for most GWAS approaches^22^), and any additional principal components from the first 20 that were nominally associated (*P* < .05) with the GWAS phenotype in a logistic regression. To obtain relatively independent index SNPs, linkage disequilibrium (LD) clumping was performed in PLINK^23^ (*r*^2^ < 0.1; *P* < 1 × 10^−4^; window size, <3 MB) for each GWAS using a reference panel of 1000 randomly selected individuals with confirmed European ancestry in the UK Biobank. Functional annotation was conducted using FUMA^24^ and LDSC^25^ was used to calculate the LD score intercept and heritability on the observed scale using the summary statistics from each psychotic experience GWAS.

#### Validation Analyses in the Avon Longitudinal Study of Parents and Children Cohort

To assess the reproducibility of the psychotic experience GWAS, we used the summary statistics from the GWAS of any psychotic experience to target psychotic experiences in the Avon Longitudinal Study of Parents and Children (ALSPAC) cohort,^26,27^ which have been previously described.^11,28^ The psychotic experience PRS was generated for each ALSPAC participant using the widely used method^29^ and logistic regression was used to test for an association between PRS and psychotic experiences reported at 12 and 18 years of age (eMethods 3 in the Supplement).

### Genetic Correlations

LDSC^25,30^ was used to calculate the genetic correlation between the GWAS of any psychotic experience and other psychiatric and personality traits. External GWAS data sets (that did not include data from the UK Biobank where possible) used to generate the correlations included schizophrenia,^31^ bipolar disorder,^32^ major depressive disorder,^33^ attention-deficit/hyperactivity disorder (ADHD),^34^ autism spectrum disorder,^35^ neuroticism,^36^ and intelligence.^37^ A Bonferroni correction was applied to control for multiple testing.

### Polygenic Risk Scores

PRSs were generated to investigate additional psychotic experience phenotypes that were not sufficiently powered for genetic correlation analyses. We selected the same summary statistics used for genetic correlations to create the risk scores for UK Biobank participants using the method described by the Psychiatric Genomics Consortium^29^ and detailed in eMethods 4 in the Supplement. The intelligence GWAS summary statistics used in this study specifically excluded participants from the UK Biobank (74 214 individuals remaining). None of the other training sets included the UK Biobank as a contributing sample, although we were not able to test for duplicates at the genotype level and so cannot rule duplicates out. Given this potential for sample overlap with the training sets, PRS results should be treated with a degree of caution and are used primarily to support and extend findings from genetic correlation analysis (which allows for overlapping samples). The primary analysis used standardized scores generated from SNPs with a discovery sample *P* value threshold of *P* ≤ .05, but associations at 10 other *P* value thresholds were also tested. A logistic regression model was used to test the association of each PRS with various psychotic experience phenotypes, covarying for the first 5 principal components and genotyping array.

### Copy Number Variation

Copy number variation (CNV) calling for the UK Biobank has been described in detail elsewhere^38^ and is detailed in eMethods 5 in the Supplement. We compared carrier status of rare CNVs previously associated with schizophrenia^39^ and neurodevelopmental disorders more widely^40^ with the 3 primary psychotic experience phenotypes used for GWASs. Association analyses were carried out using logistic regression and included age, sex, and genotyping array as covariates.

## Results

A total of 7803 (5.0%; 60.0% women and 40.0% men; mean [SD] age, 62.7 [7.7] years) individuals from the UK Biobank who completed the MHQ reported at least 1 psychotic experience, 3012 individuals rated the psychotic experience as distressing, and a total of 4388 individuals reported multiple occurrences of at least 1 psychotic experience. A total of 147 461 individuals (56.0% women and 44.0% men; mean [SD] age, 64.1 [7.6] years) who reported no psychotic experiences constituted the comparison group for our association analyses. The mean (SD) age of the first psychotic experience was 31.6 (17.6) years (eFigure 4 in the Supplement), with 2341 of 6654 (35.2%) first occurring before the age of 20 years or for as long as the participant could remember, 2137 (32.1%) between the ages of 20 and 39 years, and 2176 (32.7%) between the ages of 40 and 76 years. We excluded 198 individuals who had a diagnosis of schizophrenia, 818 with bipolar disorder, and 346 with other psychotic disorders from all genetic analyses (detailed in eMethods 1 in the Supplement).

### Genome-Wide Association Studies

**Figure 1.**
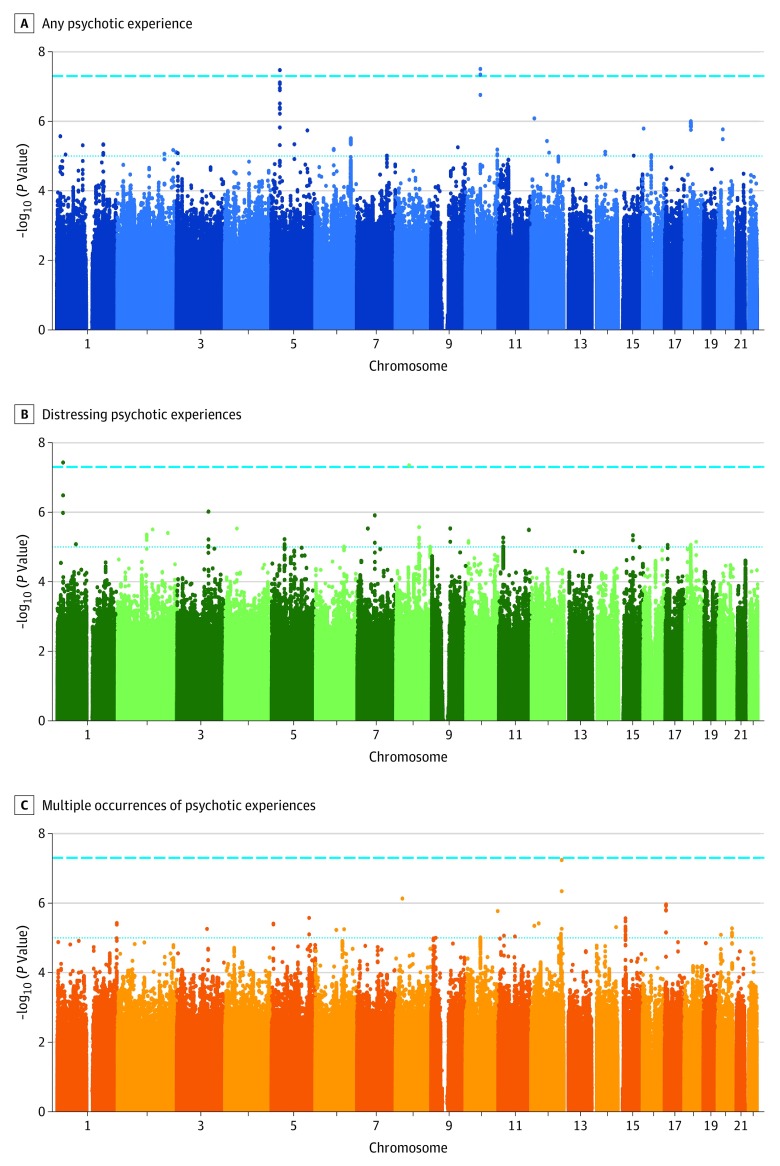
Manhattan Plot for Genome-Wide Association Study Analyses of Any Psychotic Experience, Distressing Psychotic Experiences, and Multiple Occurrences of Psychotic Experiences Dashed lines represent the genome-wide significance level of *P* < 5 × 10^−8^, and dotted lines represent *P* < 1 × 10^−5^.

The GWAS of any psychotic experience in 6123 cases and 121 843 controls (after quality control; exclusions detailed in eMethods 6 in the Supplement) identified 2 variants that were associated at the genome-wide significance level of *P* < 5 × 10^−8^ (Figure 1, Table 1; λ_GC_ = 1.05, LD score regression intercept = 1.00): rs10994278, an intronic variant within Ankyrin-3 (*ANK3* [GenBank NM_020987]) (odds ratio [OR], 1.16; 95% CI, 1.10-1.23; *P* = 3.06 × 10^−8^), and intergenic variant rs549656827 (OR, 0.61; 95% CI, 0.50-0.73; *P* = 3.30 × 10^−8^).

**Table 1.** Genome-Wide Significant Associations^a^

GWAS Phenotype	Single-Nucleotide Polymorphism	Chromosome	Base Position	A1	OR (95% CI)	*P* Value	Position	Nearest Gene
Any PE	rs10994278	10	62009219	T	1.16 (1.10-1.23)	3.06 × 10^−8^	Intronic	*ANK3*
Any PE	rs549656827	5	35349428	G	0.61 (0.50-0.73)	3.30 × 10^−8^	Intergenic	*PRLR*
Distressing PE	rs75459873	1	24256312	G	0.66 (0.56-0.78)	3.78 × 10^−8^	Intronic	*CNR2*
Distressing PE	rs3849810	8	53009606	A	1.22 (1.13-1.31)	4.55 × 10^−8^	Intergenic	*ST18*

Abbreviations: A1, risk allele; GWAS, genome-wide association study; OR, odds ratio; PE, psychotic experience.

^a^ Genome-wide significant associations (*P* < 5 × 10^−8^) from GWAS analyses.

A second GWAS restricting the cases to 2143 individuals with distressing psychotic experiences identified 2 genome-wide significance variants (Table 1; λ_GC_ = 1.03, LD score intercept = 1.01): rs75459873, intronic to cannabinoid receptor 2 (*CNR2* [GenBank NM_001841]) (OR, 0.66; 95% CI, 0.56-0.78; *P* = 3.78 × 10^−8^), and intergenic variant rs3849810 (OR, 1.22; 95% CI, 1.13-1.31; *P* = 4.55 × 10^−8^).

The third GWAS restricting the cases to 3337 individuals who reported multiple occurrences of psychotic experiences did not identify any associated variants at genome-wide significance (λ_GC_ = 1.03, LD score intercept = 1.00). QQ plots for each GWAS are displayed in eFigure 5 in the Supplement and LocusZoom plots for each genome-wide significant locus are provided in eFigure 6 in the Supplement.

Single-nucleotide polymorphism–based heritability estimates calculated by LDSC^25^ for the GWAS of any psychotic experience was *h*^2^ = 1.71% (95% CI, 1.02%-2.40%). The other GWAS analyses did not requirements (case n >5000 and *z* score >4^41^) for heritability or genetic correlation analyses with LDSC. None of the associated regions from the GWAS for any psychotic experience and distressing psychotic experience showed evidence of colocalization^42^ with schizophrenia,^31^ bipolar disorder,^32^ or major depressive disorder.^33^

#### Validation Analyses in ALSPAC

There was evidence of an association between the PRS calculated using the GWAS of any psychotic experience from the UK Biobank sample at the *P* value threshold of ≤.5 and definite psychotic experiences between 12 and 18 years of age in ALSPAC (OR, 1.13; 95% CI, 1.02-1.25; *R*^2^ = 0.002; *P* = .02). This finding was consistent for thresholds above *P* < .05 and when using measures from age 18 years only (eFigure 7 and eTable 1 in the Supplement). However, the psychotic experiences PRS was also associated with the presence of major depressive disorder at 18 years of age (OR, 1.19; 95% CI, 1.05-1.35; *R*^2^ = 0.004; *P* = .01).

#### Link Between Association at *CNR2* and Cannabis Use

We investigated, but found no evidence for, a mediating or moderating association of cannabis use with the association between distressing psychotic experiences and rs75459873 at the cannabinoid receptor gene *CNR2*. Cannabis use itself (UK Biobank field ID: 20453) was significantly associated with distressing psychotic experiences (OR, 1.36; 95% CI, 1.32-1.40; *P* = 9.16 × 10^−88^), but rs75459873 was not associated with cannabis use (OR, 0.99; 95% CI, 0.95-1.04; *P* = .70). Furthermore, the association between rs75459873 and distressing psychotic experiences was unchanged in a model including cannabis use as a covariate (OR, 0.62; 95% CI, 0.52-0.75) or as an interaction term (OR, 0.59; 95% CI, 0.48-0.73; *P* = .31 for interaction).

### Genetic Correlations

**Figure 2.**
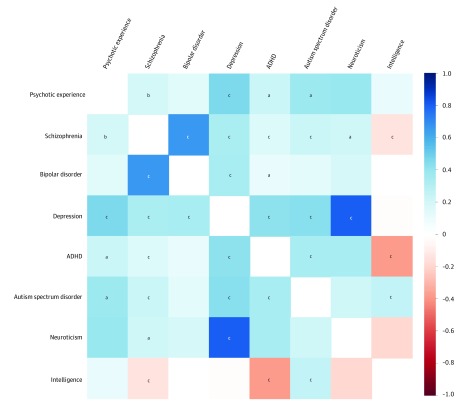
Genetic Correlation Analysis Color corresponds to the strength of the correlation, and the letters (a, b, and c) correspond to the statistical significance of the correlation. Positive correlations are shown in blue and negative correlations in red. ADHD indicates attention deficit/hyperactivity disorder. ^a^*P* < 6.25 × 10^−3^. ^b^*P* < 1.00 × 10^−4^. ^c^*P* < 1.00 × 10^−5^.

Significant genetic correlations (*r_g_*) were observed between any psychotic experience and major depressive disorder (*r_g_* = 0.46; *P* = 4.64 × 10^−11^), autism spectrum disorder (*r_g_* = 0.39; *P* = 1.68 × 10^−4^), ADHD (*r_g_* = 0.24; *P* = 4.61 × 10^−3^), and schizophrenia (*r_g_* = 0.21; *P* = 7.29 × 10^−5^). Figure 2 displays these genetic correlations and eTable 2 in the Supplement details the full results. The other psychotic experience GWASs did not meet requirements^41^ for genetic correlation analysis.

### PRS Analysis

**Figure 3.**
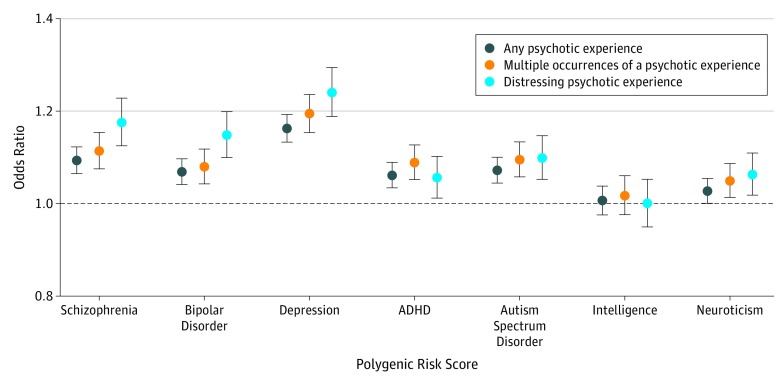
Polygenic Risk Score (PRS) Analysis The x-axis refers to the PRS tested (schizophrenia, bipolar disorder, major depressive disorder, attention deficit/hyperactivity disorder [ADHD], autism spectrum disorder, neuroticism, and intelligence), and the y-axis represents the odds ratio (OR). Points display the OR and 95% CIs (error bars) for each PRS (*P* < .05 single-nucleotide polymorphism [SNP] inclusion threshold) regressed against each psychotic experience phenotype. Plots displaying multiple *P* value SNP inclusion thresholds are shown in eFigures 8 and 9 in the Supplement.

We found evidence of a weak association between any psychotic experience and genetic liability, indicated by PRS at a *P* < .05 threshold for SNP inclusion, for schizophrenia (OR, 1.09; 95% CI, 1.06-1.12; adjusted *R*^2^ = 0.001; *P* = 2.96 × 10^−11^), major depressive disorder (OR, 1.16; 95% CI, 1.13-1.19; *R*^2^ = 0.003; *P* = 1.48 × 10^−30^), bipolar disorder (OR, 1.07; 95% CI, 1.04-1.10; *R*^2^ = 0.001; *P* = 5.11 × 10^−7^), ADHD (OR, 1.06; 95% CI, 1.03-1.09; *R*^2^ = 0.0005; *P* = 5.73 × 10^−6^), and autism spectrum disorder (OR, 1.07; 95% CI, 1.04-1.10; *R*^2^ = 0.001; *P* = 1.34 × 10^−5^). These associations were stronger for distressing psychotic experiences (Figure 3; eTable 2 in the Supplement) and consistent across most *P* value thresholds (eFigures 8 and 9 in the Supplement). We also considered individual psychotic symptoms and found that PRSs for schizophrenia, bipolar disorder, depression, and ADHD were more strongly associated with delusions of persecution than with the other psychotic symptoms (eTable 3 and eFigures 10-12 in the Supplement). The association with psychotic experience phenotypes for bipolar disorder PRS and major depressive disorder PRS remained significant in analyses controlling for schizophrenia PRS (eTable 4 in the Supplement). The association with major depressive disorder PRS remained significant when individuals with a diagnosis of depression were removed (eTable 5 in the Supplement). Given the potential for sample overlap with the training sets, PRS findings should be treated with a degree of caution.

### Copy Number Variation

Individuals reporting distressing psychotic experiences in particular had an increased burden of CNVs previously associated with schizophrenia (OR, 2.04; 95% CI, 1.39-2.98; *P* = 2.49 × 10^−4^) and neurodevelopmental disorders (OR, 1.75; 95% CI, 1.24-2.48; *P* = 1.41 × 10^−3^). There was evidence of an increased burden of these CNVs in individuals reporting any psychotic experience, but not multiple occurrences of psychotic experiences (Table 2).

**Table 2.** Association of Psychotic Experience Phenotypes With CNVs^a^

Phenotype	Rate, No./Total No. (%)		OR (95% CI)	*P* Value
	Case	Control		
Schizophrenia CNVs				
Any PE	59/5829 (1.0)	572/115807 (0.5)	1.54 (1.18-2.01)	1.60 × 10^−3^
Distressing PE	28/2046 (1.4)	572/115807 (0.5)	2.04 (1.39-2.98)	2.49 × 10^−4^
Multiple occurrence of PE	28/3177 (0.9)	572/115807 (0.5)	1.34 (0.91-1.94)	.14
Neurodevelopmental disorder CNVs				
Any PE	83/5829 (1.4)	1058/115807 (0.9)	1.54 (1.23-1.92)	1.89 × 10^−4^
Distressing PE	34/2046 (1.7)	1058/115807 (0.9)	1.75 (1.24-2.48)	1.41 × 10^−3^
Multiple occurrence of PEs	38/3177 (1.2)	1058/115807 (0.9)	1.30 (0.92-1.78)	.14

Abbreviations: CNV, copy number variant; OR, odds ratio; PE, psychotic experience.

^a^ All schizophrenia-associated CNVs are also included in neurodevelopmental disorders.

## Discussion

We conducted the largest genetic-association study of psychotic experiences, to our knowledge, using the population-based UK Biobank sample and found evidence of a shared genetic liability between psychotic experiences and several psychiatric disorders, which included, but was not specific to, schizophrenia. Genetic correlation analysis identified significant genetic correlations between psychotic experiences and major depressive disorder (*r_g_* = 0.46), autism spectrum disorder (*r_g_* = 0.39), ADHD (*r_g_* = 0.24), and schizophrenia (*r_g_* = 0.21). Polygenic risk score analyses identified associations between psychotic experiences and genetic liability for schizophrenia, major depressive disorder, bipolar disorder, ADHD, and autism spectrum disorder, and we found particular enrichment of these PRS scores in distressing psychotic experiences and for delusions of persecution. However, given the possibility of sample overlap between the UK Biobank and the training sets, the PRS findings should be treated with caution. The mechanisms underlying the high genetic correlation between depression and psychotic experiences cannot be discerned from our analysis, but one possibility is that the psychotic experiences have arisen in the context of mood-related changes, consistent with the strong associations between psychotic experiences and depressive symptoms observed in population-based studies.^43^ Nonetheless, when individuals with a lifetime history of depression were excluded from PRS analyses, the association remained, indicating that the findings are not wholly attributable to depression.

We found an increased burden of CNVs previously associated with schizophrenia and neurodevelopmental disorders more widely in individuals with any psychotic symptoms and distressing psychotic symptoms, although the association was stronger for those with distressing psychotic experiences. All schizophrenia-associated CNVs are also associated with neurodevelopmental disorders such as intellectual disability and autism spectrum disorder; in fact, penetrance is higher in individuals with these disorders.^39^ Furthermore, CNVs in the UK Biobank have been associated with a range of outcomes, including cognitive performance^38,44^ and depression,^45^ adding strength to our findings of a lack of specificity for genetic risk of psychotic experiences.

Several studies have demonstrated that psychopathologic conditions in the population are best described by a bifactor model with a common latent trait as well as specific traits, and that psychotic experiences index the more severe end of the common or shared trait.^46,47^ Our findings of nonspecificity of genetic risk for psychotic experiences with risk for other disorders are consistent with those of previous studies.^7,8,11,12^ Nonetheless, despite lacking specificity, our results suggest that incorporating questions about distress to self-reported assessments of psychotic experiences may allow a more valid identification of experiences that index liability of schizophrenia and major mental health disorders.

In the largest GWAS of psychotic experiences to date, we identified 4 genome-wide significant loci. However, consistent with other studies,^7,11^ the heritability estimate (1.71%) was low and, given that the variance explained in our PRS analysis was also low, the findings suggest that understanding the genetics of psychotic experiences is unlikely to have an important effect on understanding the genetics of schizophrenia specifically.

The primary GWAS findings are related to intronic variants in *ANK3* and *CNR2*. The GWAS of any psychotic experience identified 2 significant loci, the most significant of which was indexed by rs10994278, an intronic variant to *ANK3* (OR, 1.16; 95% CI, 1.10-1.23; *P* = 3.06 × 10^−8^). The *ANK3* gene encodes ankyrin-G, a protein that has been shown to regulate the assembly of voltage-gated sodium channels and is essential for normal synaptic function.^48^ *ANK3* is one of strongest and most replicated genes for bipolar disorder,^32^ and variants within *ANK3* have also been associated in the Psychiatric Genomics Consortium cross-disorder GWAS,^49^ and in a rare variant analysis of autism spectrum disorder.^50^

The GWAS of distressing psychotic experiences also identified 2 significant loci, the most significant of which was indexed by rs75459873, an intronic variant to *CNR2* (OR, 0.66; 95% CI, 0.56-0.78; *P* = 3.78 × 10^−8^). *CNR2* encodes for CB2, 1 of 2 well-characterized cannabinoid receptors (CB1 being the other). Several lines of evidence have implicated the endocannabinoid system in psychiatric disorders, including schizophrenia^51,52^ and depression.^53^ The main psychoactive agent of cannabis, Δ^9^-tetrahydrocannabinol, can cause acute psychotic symptoms and cognitive impairment.^54^ Given that cannabis use is strongly associated with psychotic experiences, we tested, but found no evidence for, a mediating or moderating effect of cannabis use on the association of rs75459873 and distressing psychotic experiences. However, while no evidence was found in this study, a mediating effect of cannabis use cannot be ruled out given the relatively low power of such analyses and the potential measurement error in cannabis use assessed via lifetime self-report. Independent replication of genetic loci identified in this study will be required to further understand their role in subclinical psychotic experiences.

### Strengths and Limitations

Strengths of this study include the large sample size (approximately 10 times that of previous studies), the use of an adult cohort, and the use of multiple psychotic experience phenotypes, which increase confidence in our findings. This study also has some limitations. Although we used all available information to identify and remove individuals with a psychotic disorder, it remains possible that some individuals were not identified and remained in the analysis. A further limitation of this study relates to the retrospective measurement of lifetime psychotic experiences by self-report from an online questionnaire, as this increases the likelihood of measurement error. A further limitation is the evidence of a “healthy volunteer” selection bias for the participants recruited to the UK Biobank and the sample cannot be therefore considered representative of the general population.^55^ We also found that the participants who completed the MHQ had significantly higher intelligence and lower schizophrenia, depression, and neuroticism PRS compared with UK Biobank participants who did not complete the MHQ (eTable 6 in the Supplement). Last, we were not able to entirely deduplicate the UK Biobank individuals from all the external data sets used for the PRS analysis; thus, these findings should be treated with caution.

## Conclusions

In the largest GWAS of psychotic experiences from the population-based UK Biobank sample, we found support for a shared genetic liability between psychotic experiences and several psychiatric disorders including schizophrenia, major depressive disorder, bipolar disorder, and neurodevelopmental disorders, indicating that psychotic experiences are not specifically associated with schizophrenia, but rather with a general risk for mental health disorders.

## Notes

Supplement. **eAppendix.** Unusual and Psychotic Experience Questions in UK Biobank **eMethods 1.** Defining Schizophrenia, Bipolar Disorder and Psychotic Disorder **eMethods 2.** Defining European Genetic Ancestry **eMethods 3.** Replication in ALSPAC Cohort **eMethods 4.** Calculation of Polygenic Risk Scores in UK Biobank **eMethods 5.** CNV Calling **eMethods 6.** Individuals Excluded **eFigure 1**. Psychotic Experience Phenotypes **eFigure 2**. Principal Component Analysis **eFigure 3**. Principal Component Analysis Post-Exclusions **eFigure 4**. Age of Onset of Psychotic Experiences **eFigure 5**. QQ Plots of Psychotic Experience GWAS **eFigure 6**. LocusZoom Plots of Genome-Wide Significant Loci **eFigure 7**. PRS Analysis of Psychotic Experience Symptoms in ALSPAC **eFigure 8**. PRS Analysis of Psychotic Experience Phenotypes **eFigure 9**. PRS Analysis of Psychotic Experience Phenotypes **eFigure 10.** PRS Analysis of Psychotic Experience Symptoms **eFigure 11.** PRS Analysis of Psychotic Experience Symptoms **eFigure 12.** PRS Analysis of Psychotic Experience Symptoms **eTable 1.** Validation of Psychotic Experience GWAS in ALSPAC Cohort **eTable 2.** Genetic Correlations **eTable 3.** Polygenic Risk Score Analysis **eTable 4.** Polygenic Risk Score Analysis—Covarying for Schizophrenia PRS **eTable 5.** Depression Polygenic Risk Score Analysis—Removing Individuals With Depression **eTable 6.** Association of Neuropsychiatric Polygenic Risk Scores With Completion of the Mental Health Questionnaire
