# Supplementary material for: Association of Genetic Liability to Psychotic Experiences With Neuropsychotic Disorders and Traits
Source: JAMA Psychiatry. 2019 Sep 25;76(12):1256–65. doi: 10.1001/jamapsychiatry.2019.2508 (PMC6764002; doi:10.1001/jamapsychiatry.2019.2508)
Supplement: Supplement. — eAppendix. Unusual and Psychotic Experience Questions in UK Biobank eMethods 1. Defining Schizophrenia, Bipolar Disorder and Psychotic Disorder eMethods 2. Defining European Genetic Ancestry eMethods 3. Replication in ALSPAC Cohort eMethods 4. Calculation of Polygenic Risk Scores in UK Biobank eMethods 5. CNV Calling eMethods 6. Individuals Excluded eFigure 1. Psychotic Experience Phenotypes eFigure 2. Principal Component Analysis eFigure 3. Principal Component Analysis Post-Exclusions eFigure 4. Age of Onset of Psychotic Experiences eFigure 5. QQ Plots of Psychotic Experience GWAS eFigure 6. LocusZoom Plots of Genome-Wide Significant Loci eFigure 7. PRS Analysis of Psychotic Experience Symptoms in ALSPAC eFigure 8. PRS Analysis of Psychotic Experience Phenotypes eFigure 9. PRS Analysis of Psychotic Experience Phenotypes eFigure 10. PRS Analysis of Psychotic Experience Symptoms eFigure 11. PRS Analysis of Psychotic Experience Symptoms eFigure 12. PRS Analysis of Psychotic Experience Symptoms eTable 1. Validation of Psychotic Experience GWAS in ALSPAC Cohort eTable 2. Genetic Correlations eTable 3. Polygenic Risk Score Analysis eTable 4. Polygenic Risk Score Analysis—Covarying for Schizophrenia PRS eTable 5. Depression Polygenic Risk Score Analysis—Removing Individuals With Depression eTable 6. Association of Neuropsychiatric Polygenic Risk Scores With Completion of the Mental Health Questionnaire [file jamapsychiatry-76-1256-s001.pdf]

Supplementary Online Content

Legge SE, Jones HJ, Kendall KM, et al. Association of genetic liability to psychotic experiences with neuropsychotic disorders and traits. *JAMA Psychiatry.* Published online September 25, 2019. doi:10.1001/jamapsychiatry.2019.2508

eAppendix. Unusual and Psychotic Experience Questions in UK Biobank
eMethods 1. Defining Schizophrenia, Bipolar Disorder and Psychotic Disorder
eMethods 2. Defining European Genetic Ancestry
eMethods 3. Replication in ALSPAC Cohort
eMethods 4. Calculation of Polygenic Risk Scores in UK Biobank
eMethods 5. CNV Calling
eMethods 6. Individuals Excluded
eFigure 1. Psychotic Experience Phenotypes
eFigure 2. Principal Component Analysis
eFigure 3. Principal Component Analysis Post-Exclusions
eFigure 4. Age of Onset of Psychotic Experiences
eFigure 5. QQ Plots of Psychotic Experience GWAS
eFigure 6. LocusZoom Plots of Genome-Wide Significant Loci
eFigure 7. PRS Analysis of Psychotic Experience Symptoms in ALSPAC
eFigure 8. PRS Analysis of Psychotic Experience Phenotypes
eFigure 9. PRS Analysis of Psychotic Experience Phenotypes
eFigure 10. PRS Analysis of Psychotic Experience Symptoms
eFigure 11. PRS Analysis of Psychotic Experience Symptoms
eFigure 12. PRS Analysis of Psychotic Experience Symptoms
eTable 1. Validation of Psychotic Experience GWAS in ALSPAC Cohort
eTable 2. Genetic Correlations
eTable 3. Polygenic Risk Score Analysis
eTable 4. Polygenic Risk Score Analysis—Covarying for Schizophrenia PRS
eTable 5. Depression Polygenic Risk Score Analysis—Removing Individuals With Depression
eTable 6. Association of Neuropsychiatric Polygenic Risk Scores With Completion of the Mental Health Questionnaire

This supplementary material has been provided by the authors to give readers additional information about their work.

© 2019 Legge SE et al. *JAMA Psychiatry*.

eAppendix. Unusual and Psychotic Experience Questions in UK Biobank

The following questions are from Section F of the Mental Health Questionnaire (MHQ) administered via web-based questionnaire for UK Biobank participants.

Q. No	Question	Responses
INTRO	The next set of questions is about unusual experiences that you may have had, like seeing visions or hearing voices. We believe that these things may be quite common, but we don't know for sure. So please take your time and think carefully before answering.	
F1	Did you ever see something that wasn't really there that other people could not see?<br><br>Please do not include any times when you were dreaming or half-asleep or under the influence of alcohol or drugs.	[Choose one from]<br>- 01 Yes<br>- 00 No<br>- NA Do not know<br>- DA Prefer not to answer
F1a	About how many times in your life did this happen (when you were not dreaming, not half-asleep, and not under the influence of alcohol or drugs)?	FBOX1: Integer box 1 – 999<br>FBOX1 & "time(s)"<br>OR<br>- 01 Too many to count<br>- NA Do not know<br>- DA Prefer not to answer
F2	Did you ever hear things that other people said did not exist, like strange voices coming from inside your head talking to you or about you, or voices coming out of the air when there was no one around?<br><br>Please do not include any times when you were dreaming or half-asleep or under the influence of alcohol or drugs.	[Choose one from]<br>- 01 Yes<br>- 00 No<br>- DA Prefer not to say<br>- NA Don't know
F2a	About how many times in your life did this happen (when you were not dreaming, not half-asleep, and not under the influence of alcohol or drugs)?	FBOX2: Integer box 1 – 999<br>FBOX2 & "time(s)"<br>OR<br>- 01 Too many to count<br>- NA Do not know<br>- DA Prefer not to answer
F3	Did you ever believe that a strange force was trying to communicate directly with you by sending special signs or signals that you could understand but that no one else could understand (for example through the radio or television)?<br><br>Please do not include any times when you were dreaming or half-asleep or under the influence of alcohol or drugs.	[Choose one from]<br>- 01 Yes<br>- 00 No<br>- NA Do not know<br>- DA Prefer not to answer
F3a	About how many times in your life did this happen (when you were not dreaming, not half-asleep, and not under the influence of alcohol or drugs)?	FBOX3: Integer box 1 – 999<br>FBOX3 & "time(s)"<br>OR<br>- 01 Too many to count<br>- NA Do not know<br>- DA Prefer not to answer
F4	Did you ever believe that that there was an unjust plot going on to harm you or to have people follow you, and which your family and friends did not believe existed?<br><br>Please do not include any times when you were dreaming or half-asleep or under the influence of alcohol or drugs.	[Choose one from]<br>- 01 Yes<br>- 00 No<br>- NA Do not know<br>- DA Prefer not to answer

© 2019 Legge SE et al. *JAMA Psychiatry*.

F4a	About how many times in your life did this happen (when you were not dreaming, not half-asleep, and not under the influence of alcohol or drugs)?	FBOX4: Integer box 1 – 999<br>FBOX4 & "time(s)"<br>OR<br>- 01 Too many to count<br>- NA Do not know<br>- DA Prefer not to answer
F5	How often did any of these experiences happen in the past 1 year (seeing a vision, hearing a voice, or believing that something strange was trying to communicate with you, or there was a plot against you)?	[Choose one from]<br>- 00 Not at all<br>- 01 Once or twice<br>- 02 Less than once a month<br>- 03 More than once a month<br>- 04 Nearly every day or daily<br>- DA Prefer not to answer
F6	How old were you (approximately) when you first had one of these experiences (seeing a vision, hearing a voice, or believing that something strange was trying to communicate with you, or there was a plot against you)?	FBOX5: Integer box 2 to current age<br>FBOX5 & "years old"<br>OR<br>- 01 As long as I can remember<br>- NA Do not know<br>- DA Prefer not to answer
F7	How distressing did you find having any of these experiences (seeing a vision, hearing a voice, or believing that something strange was trying to communicate with you, or there was a plot against you)?	[Choose one from]<br>- 00 Not distressing at all, it was a positive experience<br>- 01 Not distressing, a neutral experience<br>- 02 A bit distressing<br>- 03 Quite distressing<br>- 04 Very distressing<br>- NA Do not know<br>- DA Prefer not to answer
F8	Did you ever talk to a doctor, counselor, psychiatrist or other health professional about any of these experiences (seeing a vision, hearing a voice, or believing that something strange was trying to communicate with you, or there was a plot against you)?	[Choose one from]<br>- 01 Yes<br>- 00 No<br>- NA Do not know<br>- DA Prefer not to answer
F9	Were you ever prescribed a medication by a health professional for any of these experiences (seeing a vision, hearing a voice, or believing that something strange was trying to communicate with you, or there was a plot against you)?	[Choose one from]<br>- 01 Yes<br>- 00 No<br>- NA Do not know<br>- DA Prefer not to answer

© 2019 Legge SE et al. *JAMA Psychiatry*.

eMethods 1. Defining Schizophrenia, Bipolar Disorder and Psychotic Disorder

We searched for evidence of a diagnosis of schizophrenia, bipolar affective disorder, or a psychotic disorder from numerous sources within UK Biobank. Individuals were classed as having one of these disorders if there was any indication from any of the following sources (i) self-reported diagnosis at the assessment centre interview (UK Biobank field ID: 20002), (ii) an ICD-10 primary (UK Biobank field ID: 41202) or secondary (UK Biobank field ID: 41204) diagnosis from linked hospital records, (iii) an ICD-10 diagnosis from death records (UK Biobank field IDs: 40001 and 40002), or (iv) a self-report of a relevant diagnosis made by a health professional in the mental health questionnaire (MHQ) (UK Biobank field ID: 20544). The ICD-10 codes used for schizophrenia consisted of F20 and F25, for bipolar affective disorder F30 and F31, and for psychotic disorder F21, F22, F23, F28 and F29.

© 2019 Legge SE et al. *JAMA Psychiatry*.

eMethods 2. Defining European Genetic Ancestry

Analyses were restricted to individuals with a self-reported British and Irish ethnic background (UK Biobank field ID: 21000). The first 40 principal components supplied by UK Biobank[1] (UK Biobank field ID: 22009) were used to assess and control for population structure. Initial analyses (**eFigure 2**) showed that filtering by self-reported ancestry does not adequately control for ancestry, and so European genetic ancestry was confirmed using the 'covMCD' function in the R package 'robustbase'[2], which uses the first five principal components to compute a Minimum Covariance Determinant (MCD) estimator of location and scatter via the deterministic MCD algorithm[3]. The MCD defines a hyper-ellipsoid in a multi-dimensional space that contains the majority of points representing individuals in that group, and once this has been defined, all individuals are allocated a distance to the hyper-ellipsoid in PC space[4]. We selected individuals that were within the 90th percentile of MCD distance (**eFigure 3**).

© 2019 Legge SE et al. *JAMA Psychiatry*.

eMethods 3. Replication in ALSPAC Cohort

To assess the validity of the psychotic experience phenotype, we targeted psychotic experiences in the Avon Longitudinal Study of Parents and Children (ALSPAC) longitudinal birth cohort.

ALSPAC study population

The initial cohort consisted of 14,541 pregnant women residing in the former Avon Health Authority area with an expected delivery date between April 1991 and December 1992[5,6]. Of these initial pregnancies, there was a total of 14,676 fetuses, resulting in 14,062 live births and 13,988 children who were alive at 1 year of age. When the oldest children were approximately 7 years of age, an attempt was made to bolster the initial sample with eligible cases who had failed to join the study originally. The total sample size for analyses using any data collected after the age of 7 is therefore 15,247 pregnancies, resulting in 15,458 fetuses. Of this total sample of 15,656 fetuses, 14,973 were live births and 14,899 were alive at 1 year of age. Please note that the study website contains details of all the data that is available through a fully searchable data dictionary and variable search tool (http://www.bristol.ac.uk/alspac/researchers/our-data/). Ethical approval for the study was obtained from the ALSPAC Ethics and Law Committee and the Local Research Ethics Committees. Informed consent for the use of data collected via questionnaires and clinics was obtained from participants following the recommendations of the ALSPAC Ethics and Law Committee at the time and consent for biological samples has been collected in accordance with the Human Tissue Act (2004).

ALSPAC genetic data

Genetic data were acquired using the Illumina HumanHap550 quad genome-wide single nucleotide polymorphism (SNP) genotyping platform from 9912 participants. Individuals were excluded from further analysis based on gender mismatches, minimal or excessive heterozygosity, disproportionate levels of individual missingness (>3%), evidence of cryptic relatedness (>10% of alleles identical by descent) and being of non-European ancestry (assessed by multidimensional scaling analysis including HapMap 2 individuals). SNPs with a minor allele frequency (MAF) of < 1%, Impute2 information quality metric of < 0.8, a call rate of < 95% or evidence for violations of Hardy-Weinberg equilibrium (p-value $< 5 \times 10^{-7}$) were removed. Imputation of the target data was performed using Impute v2.2.2 against the 1000 genomes reference panel (Phase 1, Version 3; all polymorphic SNPs excluding singletons), using 2,186 reference haplotypes (including non-Europeans). Following quality control assessment and imputation and restricting to 1 young person per family, genetic data was available for 7975 ALSPAC individuals.

Generating psychotic experiences polygenic risk scores in ALSPAC

Prior to construction of polygenic risk scores (PRSs) for psychotic experiences, SNPs were removed from the analysis if they had a MAF < 0.01, an imputation quality < 0.8, or if there was allelic mismatch between samples (the alleles reported by the discovery study did not match the alleles present in the ALSPAC sample). Due to the high linkage disequilibrium (LD) within the extended major histocompatibility complex (MHC; chromosome 6: 25-34Mb) only a single SNP was included to represent this region within the analysis. Remaining SNPs were then further pruned for LD using the PLINK (v1.90)[7] --

© 2019 Legge SE et al. *JAMA Psychiatry*.

clump command to retain SNPs with an association p-value ≤ 0.5 and r2 < 0.25 within 500kb windows.

PRSs were constructed using the summary statistics from the primary GWAS of *any psychotic experience* in UK Biobank. PRSs were calculated for each ALSPAC individual using the PLINK (v1.07)[7] --score command. Scores are calculated by summing the number of reference alleles present for each SNP (0, 1 or 2) weighted by the logarithm of its odds ratio for *any psychotic experience* and standardized prior to analyses.

Our primary analysis used a score generated using a list of SNPs meeting an *any psychotic experience* GWAS p-value threshold of ≤ 0.50. To assess the robustness of our findings, analyses were then repeated using PRSs based on SNPs that were associated with *any psychotic experience* at a range of GWAS p-value thresholds (p-value ≤ $1e^{-6}$ to ≤ 0.5).

Psychotic experiences in ALSPAC

The semi-structured Psychosis-Like Symptom Interview (PLIKSi)[8,9], which draws on principles of standardized clinical examination developed for the Schedule for Clinical Assessment in Neuropsychiatry (SCAN), was used to assess psychotic experiences in ALSPAC at ages 12 and 18 years. The PLIKSi allows rating of 12 psychotic experiences including hallucinations (visual and auditory), delusions (spied on, persecution, thoughts read, reference, control, grandiosity, other) and experiences of thought interference (broadcasting, insertion and withdrawal). Any unspecified delusions elicited are also rated. Structured stem questions (e.g. “have you ever seen something or someone that other people could not see?”; “have you ever thought you were being followed or spied on?”; “Have you ever felt that thoughts are put into your mind that are not your own?”) are followed up by cross-questioning to allow the interviewer to make a decision as to whether experiences described meet SCAN criteria for a psychotic experience.

The interviewers were psychology graduates trained in assessment using the SCAN psychosis section and using the PLIKSi. Psychotic experiences were rated as not present, suspected or definitely psychotic. Unclear responses were always ‘rated down’ and symptoms were rated as definite only when a clear example was provided. At regular intervals samples of recorded interviews were also rated by a psychiatrist to ensure interviewers were rating experiences correctly. The PLIKSi shows very good inter-rater and test-retest reliability[10].

To maximise the numbers within our sample we used data from both the interviews at ages 12 and 18 years to identify individuals rated as having one or more definite psychotic experiences between ages 12 and 18 years, compared to no or only suspected psychotic experiences across this age range.

To assess the sensitivity of results, analyses were also performed using data from age 12 and age 18 years separately and using a different cut-off of definite or suspected psychotic experiences compared to no psychotic experiences at 12 or 18 years. A latent variable was also generated for psychotic experiences at age 16.5 years using ten items from the self-report Psychosis-Like Symptoms Questionnaire (PLIKSq)[11]. These ten items were rated on a 3-point scale (never; maybe; definitely) and assessed presence of hallucinations, delusions and thought interference since age 15. The psychotic experience latent variable was generated in Mplus (version 7.31) as previously reported[12].

© 2019 Legge SE et al. *JAMA Psychiatry*.

Logistic regressions were performed in Stata v15.1 to test for associations between *any psychotic experience* PRSs and ALSPAC psychotic experiences reported at age 12 and 18 years. Linear regressions were used in Mplus to test for associations between *any psychotic experience* PRSs and the latent psychotic experience trait at age 16.5 years. No principal components were included in the PRS regression analyses, as is standard for studies in this cohort due to homogenous nature of the sample[13].

© 2019 Legge SE et al. *JAMA Psychiatry*.

eMethods 4. Calculation of Polygenic Risk Scores in UK Biobank

To examine the relationship between psychotic experiences and genetic risk for schizophrenia, bipolar disorder, depression, attention deficit/hyperactivity disorder (ADHD), autism spectrum disorder, neuroticism and intelligence, PRSs were generated using the method described by the PGC[14]. External discovery datasets used to generate the PRSs included; schizophrenia[15], bipolar disorder[16], major depressive disorder[17], ADHD[18], autism spectrum disorder[19], neuroticism[20], and lastly for intelligence, summary statistics were obtained for the latest GWAS[21] excluding UK Biobank participants (n=74,214 individuals remaining, summary statistics specifically derived for this study). PRSs were calculated using imputation dosage data for each UK Biobank participant that passed GWAS QC measures using PRSice (v2)[22]. High quality SNPs were selected by applying filters for INFO > 0.9, MAF > 0.1, removing indels, and excluding the extended MHC region (25 Mb – 35 Mb). A reference panel of 1000 randomly selected UK Biobank participants was used to obtain relatively independent SNPs ($r^2 < 0.2$, window size < 500kb). The first five principal components, any additional principal components from the first 20 that were nominally associated ($p < 0.05$) with the GWAS phenotype in a logistic regression, and genotyping array, were added as covariates for each PRS generated. Scores were generated at 11 SNP p-value thresholds (5×10^{-8}, 1×10^{-7}, 5×10^{-6}, 5×10^{-5}, 5×10^{-4}, 0.005, 0.05, 0.10, 0.20, 0.50, 1).

Our primary analysis of PRS used standardised scores generated from SNPs with a GWAS discovery sample p-value threshold of $p \leq 0.05$. Statistical association analyses were conducted in R and used a regression model to test the association of each PRS with the various psychotic experience phenotypes, and covarying for the first five principal components and genotyping array. The Nagelkerke R^2 and area under the curve (AUC) values were adjusted for covariates.

© 2019 Legge SE et al. *JAMA Psychiatry*.

eMethods 5. CNV Calling

CNV calling, which has been described in detail elsewhere[23], was carried out using biallelic markers common to both genotyping platforms using PennCNV-Affy[24]. Exclusion criteria included ≥ 30 CNVs, waviness factor <-0.03 or > 0.03, call rate < 0.96 for individual samples, LRR SD > 0.35 and coverage of < 20 probes, density coverage of < 1 probe per 20,000 base pairs or a confidence score of < 10 for individual CNVs.

We compared carrier status of rare CNVs previously associated with (i) schizophrenia[25] and (ii) neurodevelopmental disorders more widely[26] (which includes all schizophrenia-associated CNVs and excludes 15q11.2 duplication because of its high frequency following our previous publication[23]) with the three primary psychotic experience phenotypes used for GWAS. Association analyses were carried out using logistic regression and included age, sex and genotyping array as covariates.

© 2019 Legge SE et al. *JAMA Psychiatry*.

eMethods 6. Individuals Excluded

The table below details the numbers excluded during each quality control step.

QC step	Comparison group	Any PE	Distressing PE	Multiple PE
1	13,429	840	360	510
2	4,603	274	97	150
3	7,511	11	6	7
4	539	555	406	384
Total remaining	121,379	6,123	2,143	3,337

Quality control step 1 consisted of excluding study individuals that (i) did not have a self-reported White British or Irish ethnicity, (ii) did not have genetic data available, or (iii) did not pass initial genetic quality control parameters (missingness). Step 2 consisted of excluding individuals that did not have European genetic ancestry as defined by principal components (see 'defining European genetic ancestry' above). Step 3 consisted of excluding related individuals and finally, step 4 excluded individuals with a schizophrenia, bipolar disorder or psychotic disorder diagnosis.

© 2019 Legge SE et al. *JAMA Psychiatry*.

eFigure 1. Psychotic Experience Phenotypes

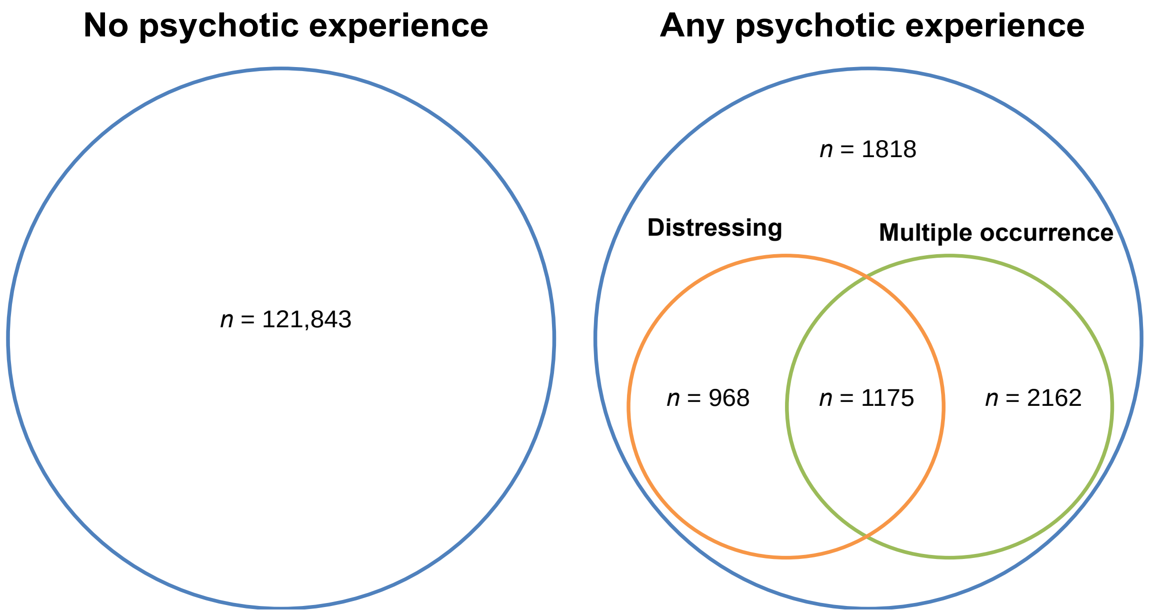

Venn diagram detailing the sample overlaps between the phenotypes used in the three psychotic experience GWAS analyses. The left hand circle represents the comparison group, who reported no psychotic experiences. The right hand circle represents the cases used in the three GWAS (i) any psychotic experience, (ii) distressing psychotic experiences, and (iii) multiple occurrences of psychotic experiences.

© 2019 Legge SE et al. *JAMA Psychiatry*.

eFigure 2. Principal Component Analysis

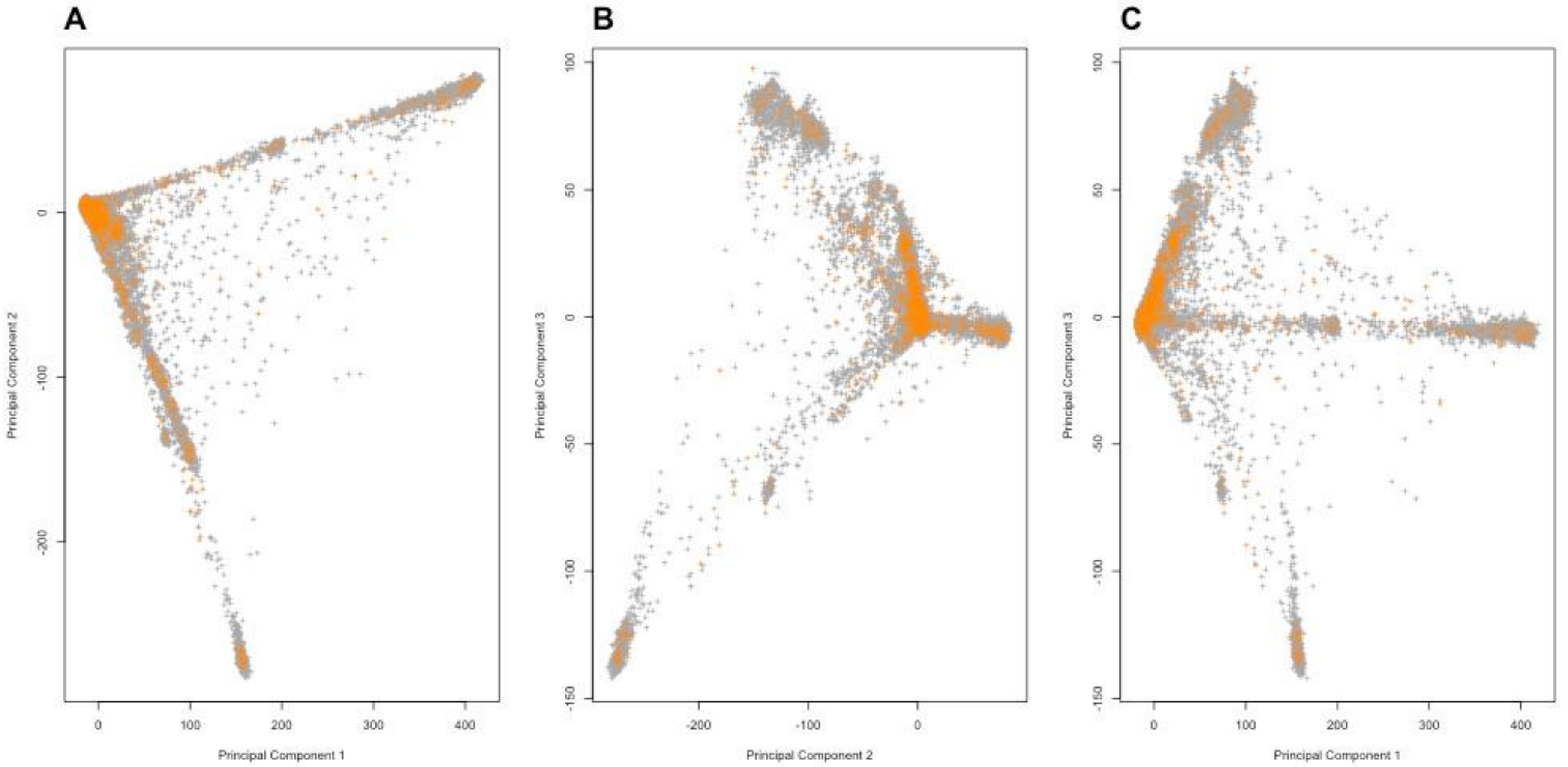

Principal component analysis. Points represent individuals who completed the MHQ. Grey points: *n*=139,975 individuals who completed MHQ but did not report a psychotic experience. Orange points: *n*=7,791 individuals reporting *any psychotic experience*. Plot A: principal component 1 vs. principal component 2 Plot B: principal component 2 vs. principal component 3. Plot C: principal component 1 vs. principal component 3.

© 2019 Legge SE et al. *JAMA Psychiatry*.

eFigure 3. Principal Component Analysis Post-Exclusions

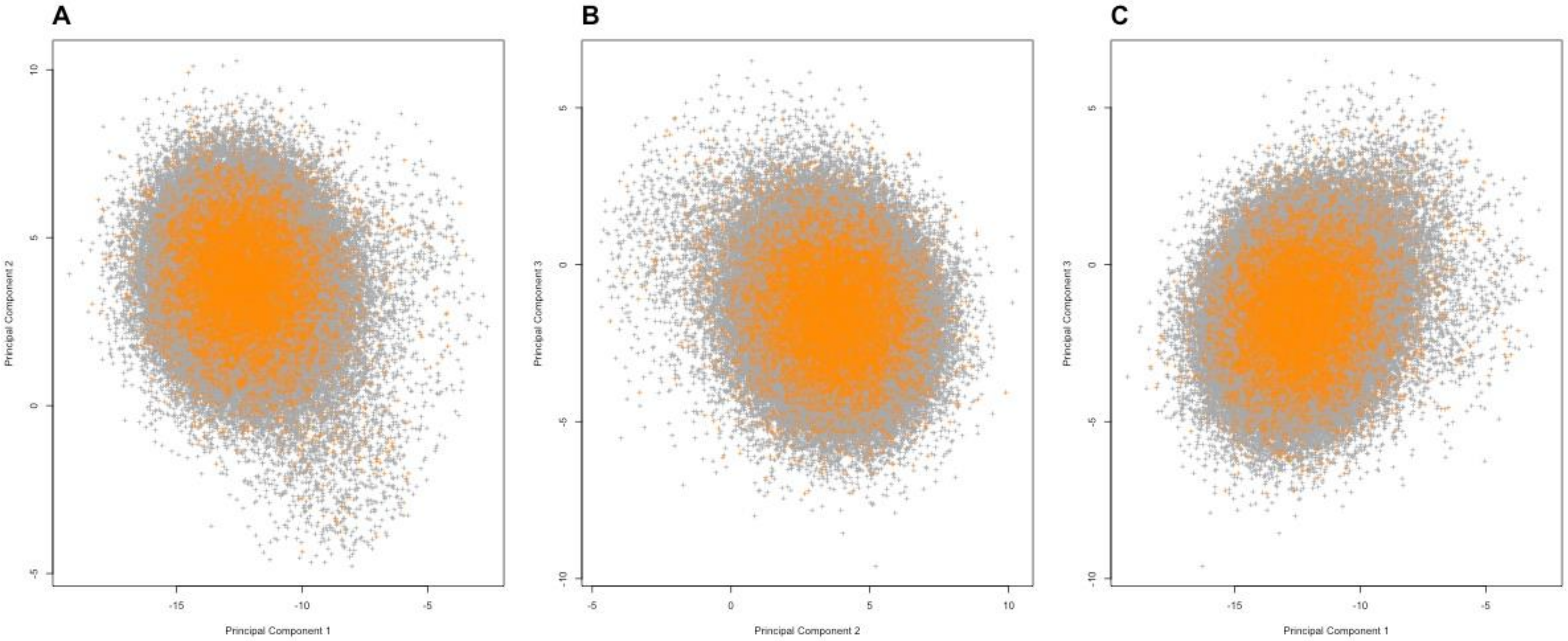

Principal component analysis after exclusions from MCD model. Points represent individuals who completed the MHQ. Grey points: individuals who completed MHQ but did not report a psychotic experience. Orange points: individuals reporting *any psychotic experience*. Plot A: principal component 1 vs principal component 2. Plot B: principal component 2 vs. principal component 3. Plot C: principal component 1 vs. principal component 3.

© 2019 Legge SE et al. *JAMA Psychiatry*.

eFigure 4. Age of Onset of Psychotic Experiences

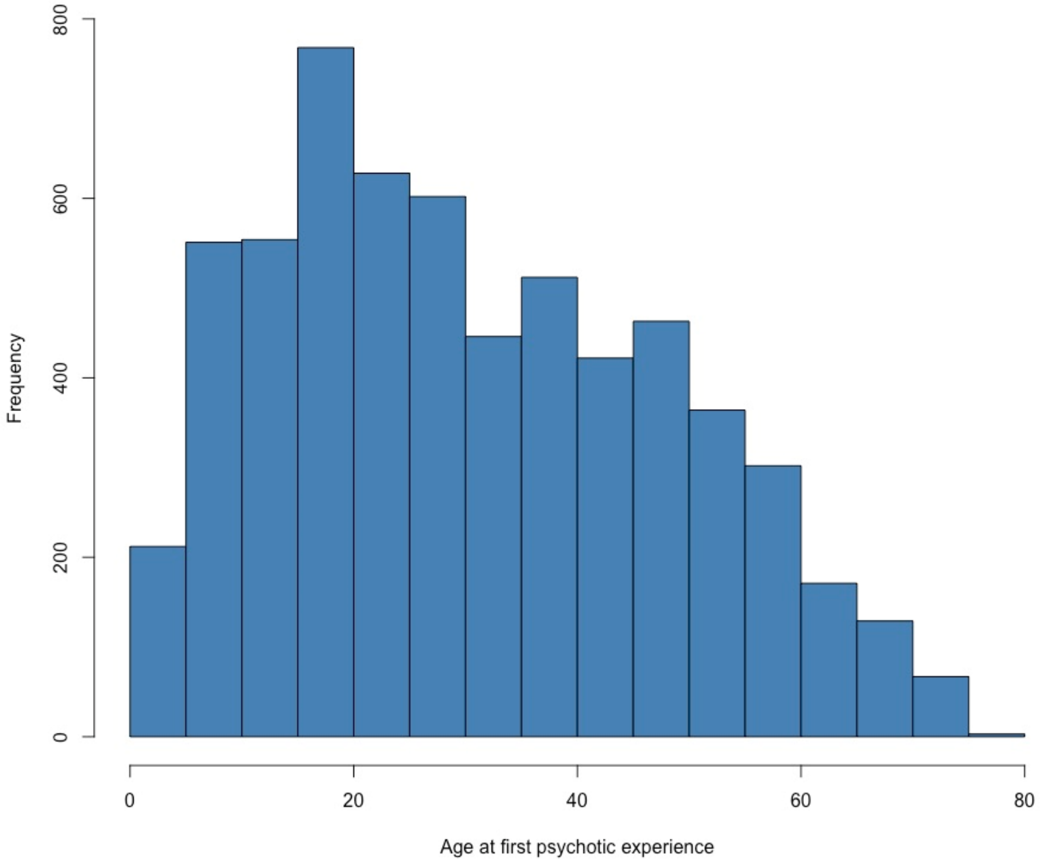

Histogram of age at first psychotic experience in UK Biobank.

© 2019 Legge SE et al. *JAMA Psychiatry*.

eFigure 5. QQ Plots of Psychotic Experience GWAS

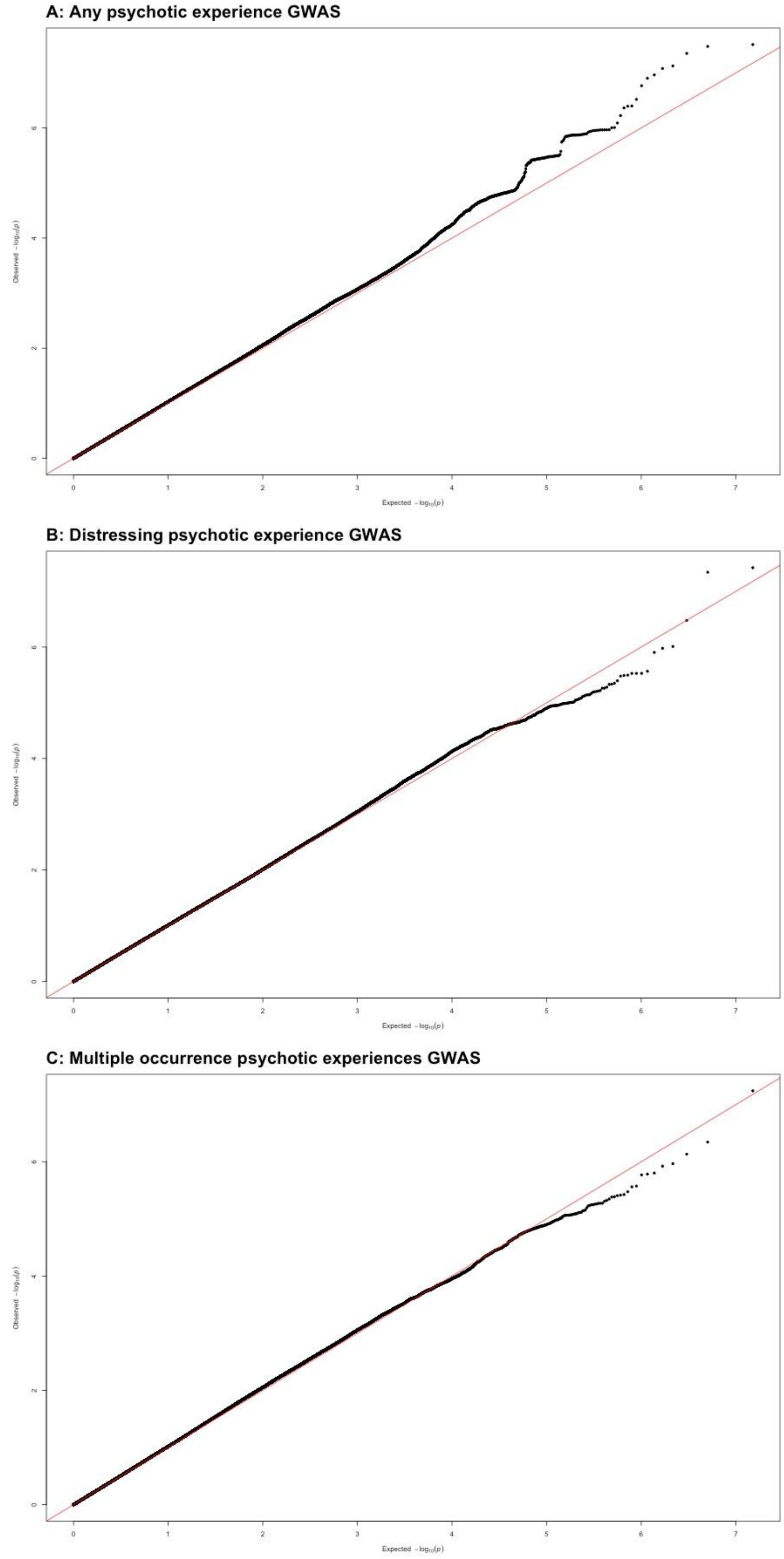

Quantile-quantile (QQ) plots of GWAS analyses. Plot A: any psychotic experience, plot B: distressing psychotic experiences, plot C: multiple occurrence psychotic experiences.

© 2019 Legge SE et al. *JAMA Psychiatry*.

eFigure 6. LocusZoom Plots of Genome-Wide Significant Loci

A

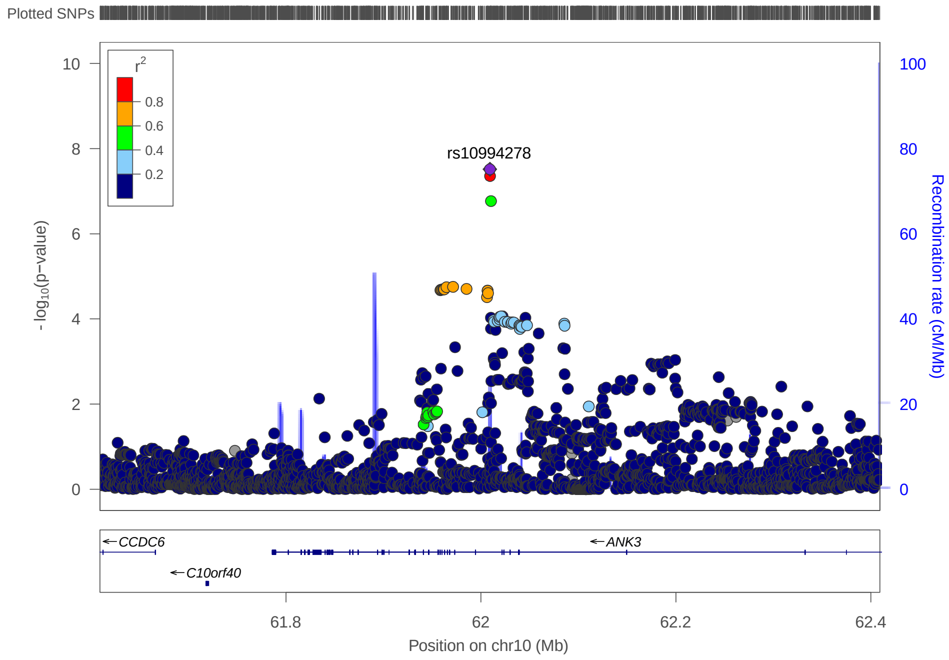

B

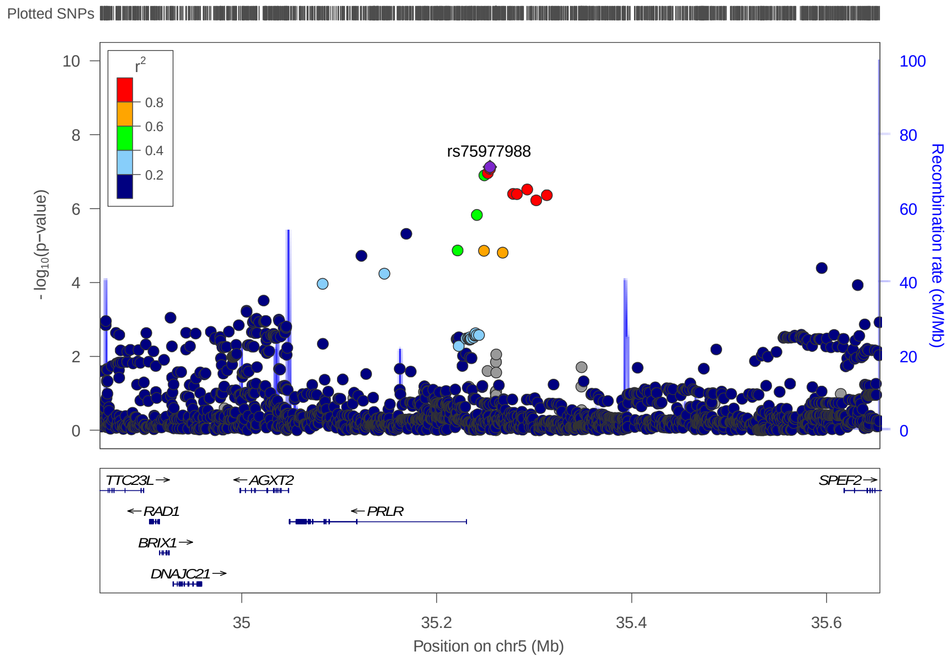

© 2019 Legge SE et al. *JAMA Psychiatry*.

C

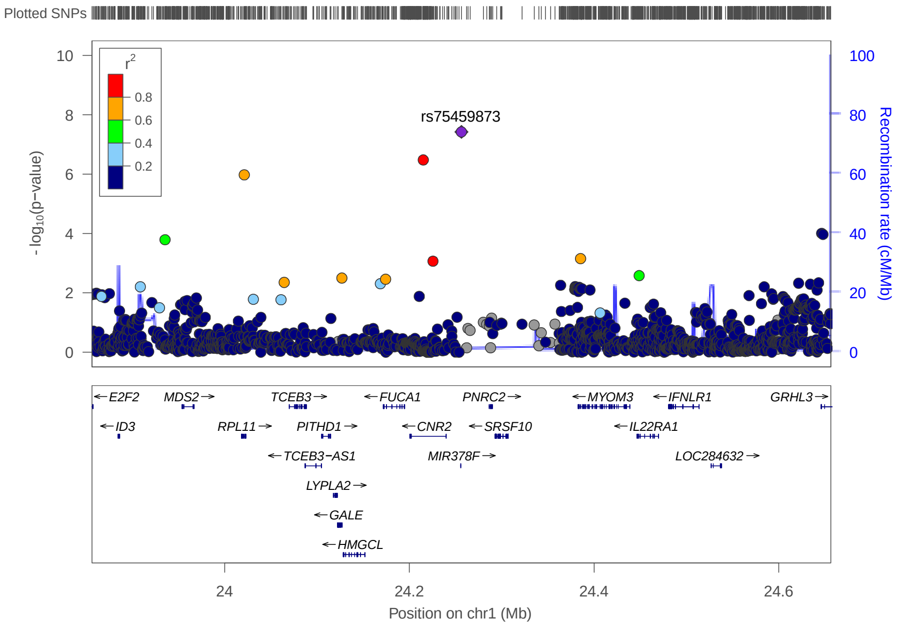

D

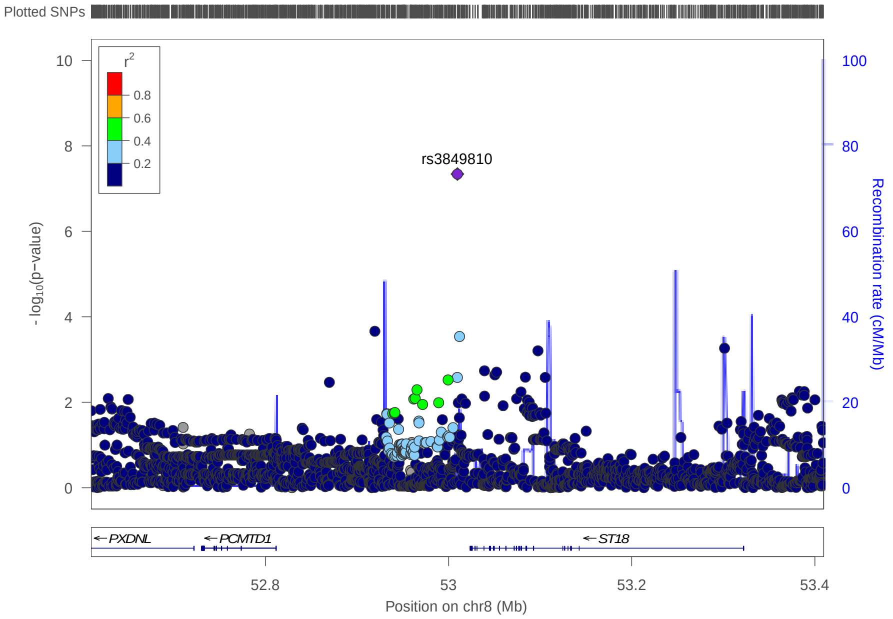

LocusZoom[27] plots for significantly associated loci from GWAS analyses; A = rs10994278 (*any psychotic experience* GWAS), B = rs75977988 (proxy for rs549656827 (D'=1), *any psychotic experience* GWAS) , C = rs75459873 (*distressing psychotic experiences* GWAS), D = rs3849810 (*distressing psychotic experiences* GWAS). Genes within the regions are shown in each lower panel and the unbroken blue line indicates the recombination rate within the region. The index SNP (with the highest P-value) for each region is shown in purple.

© 2019 Legge SE et al. *JAMA Psychiatry*.

eFigure 7. PRS Analysis of Psychotic Experience Symptoms in ALSPAC

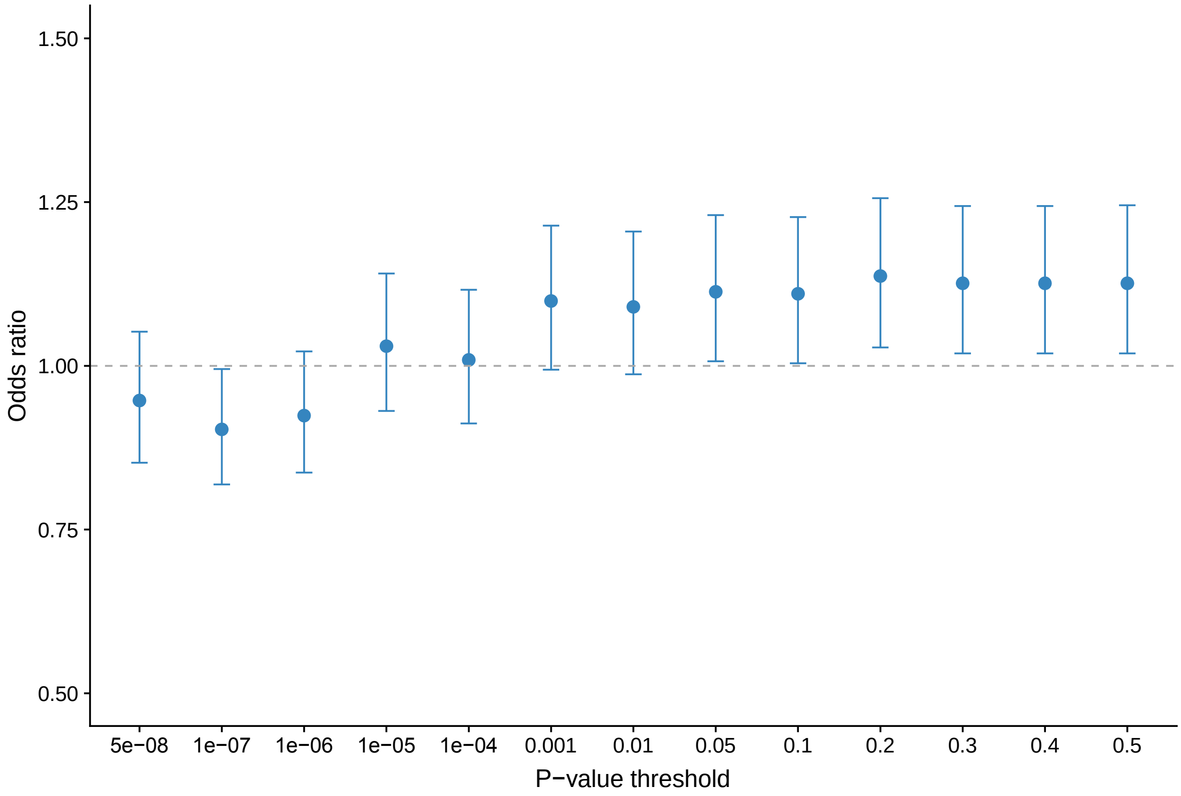

Polygenic risk score (PRS) analysis in ALSPAC cohort. PRS was created using summary statistics from the GWAS of *any psychotic experience* GWAS in UK Biobank and targeted psychotic experiences in ALSPAC (PLIKSi at 12 or 18, n = 5310, 7.76% with psychotic experiences). See **eTable 1** for full results.

© 2019 Legge SE et al. *JAMA Psychiatry*.

eFigure 8. PRS Analysis of Psychotic Experience Phenotypes

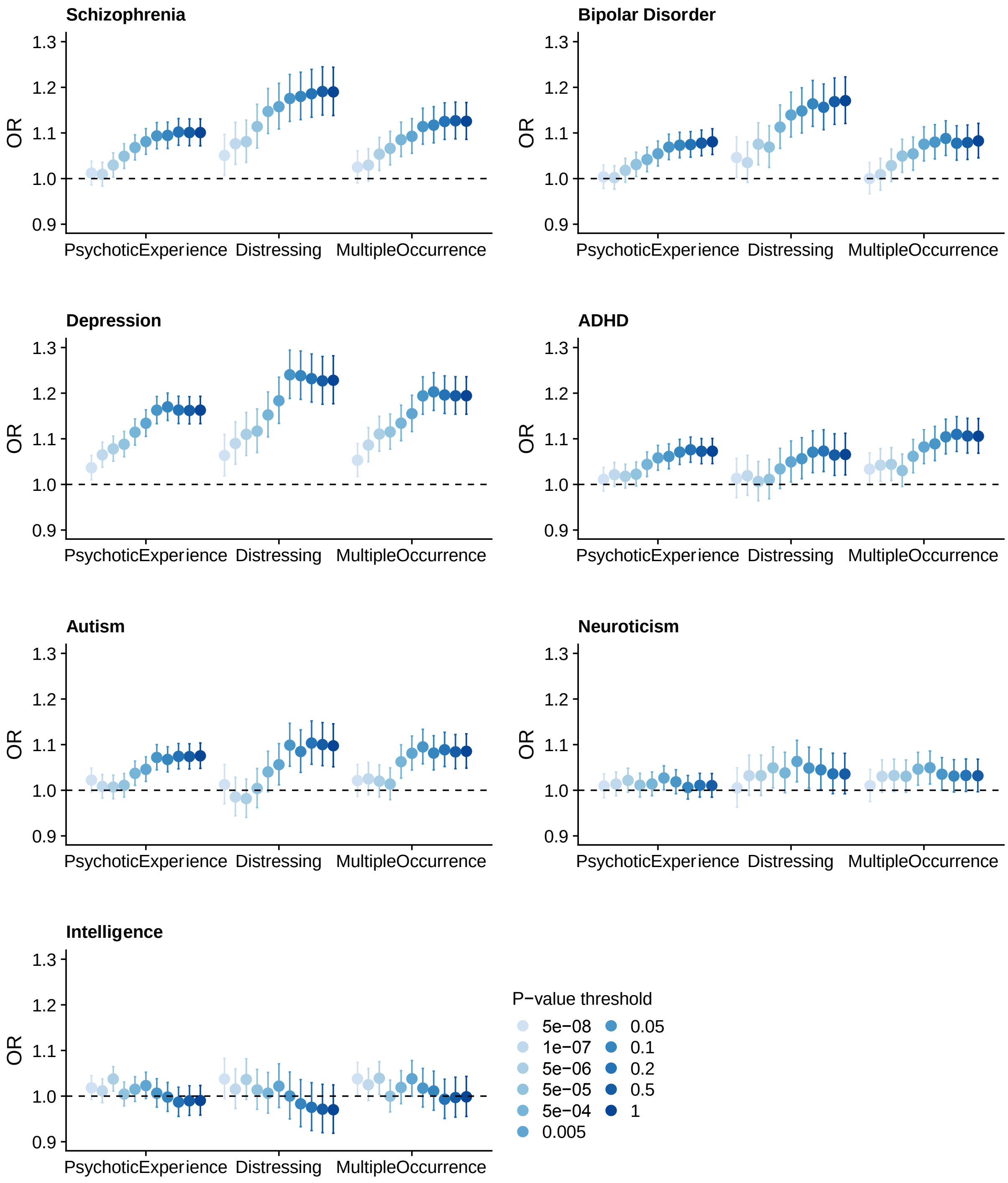

Polygenic risk score (PRS) analysis. Each plot shows results for each PRS (schizophrenia, bipolar disorder, major depressive disorder, attention deficit/hyperactivity disorder (ADHD), autism spectrum disorder, neuroticism and intelligence). The x-axis shows the psychotic experience phenotype (any psychotic experience, distressing psychotic experiences and multiple occurrence psychotic experiences). Points display the odds ratio (OR) and error bars represent the 95% confidence intervals for the OR.

© 2019 Legge SE et al. *JAMA Psychiatry*.

eFigure 9. PRS Analysis of Psychotic Experience Phenotypes

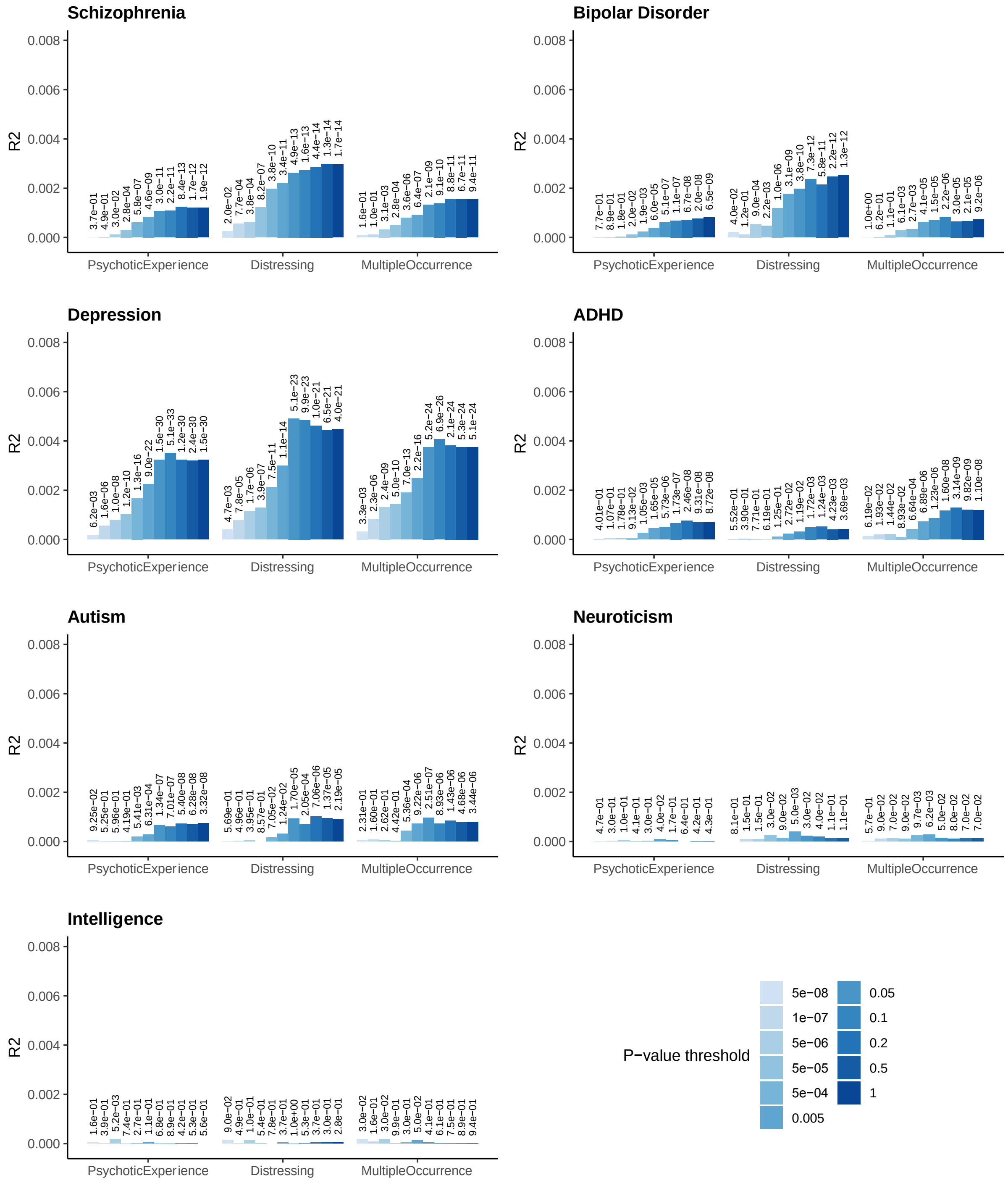

Polygenic risk score (PRS) analysis. Each plot shows results for each PRS (schizophrenia, bipolar disorder, major depressive disorder, attention deficit/hyperactivity disorder (ADHD), autism spectrum disorder, neuroticism and intelligence). The x-axis shows the psychotic experience phenotype (any psychotic experience, distressing psychotic experiences and multiple occurrence psychotic experiences). Bars represent adjusted variance explained (R^2) and association p-values are listed above.

© 2019 Legge SE et al. *JAMA Psychiatry*.

eFigure 10. PRS Analysis of Psychotic Experience Symptoms

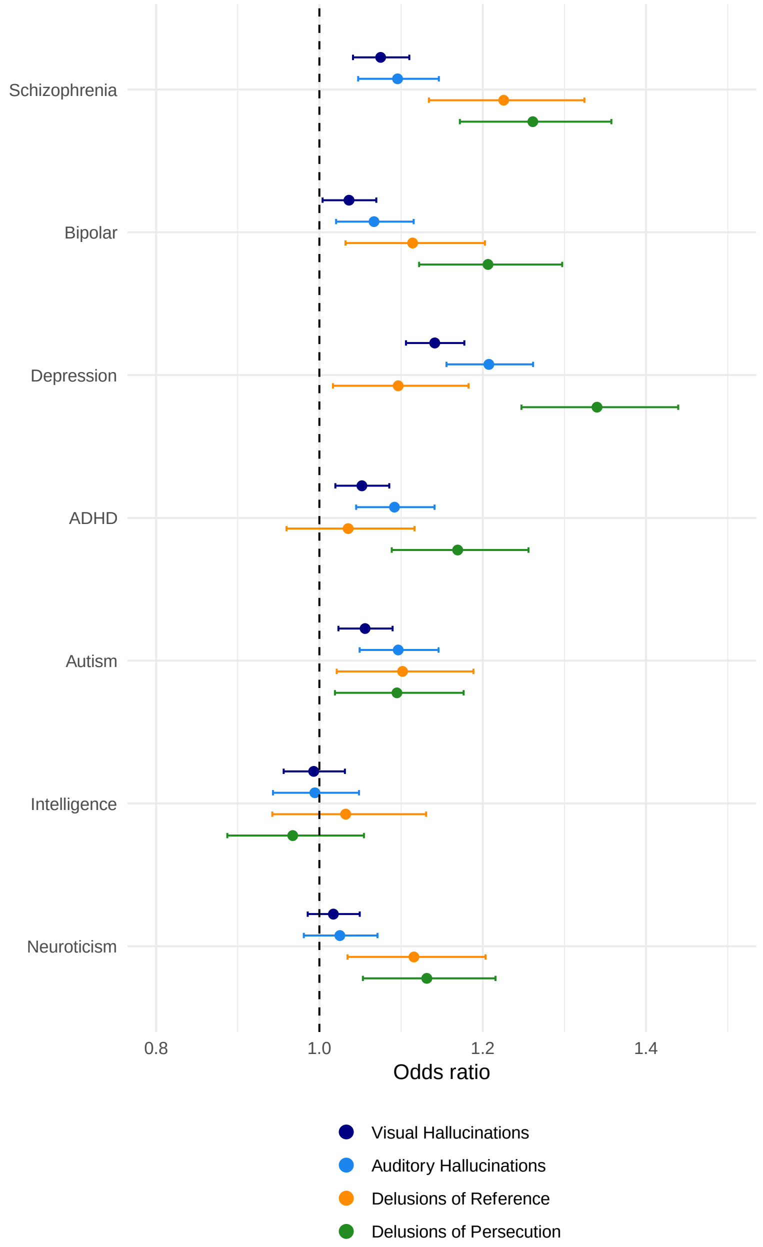

Polygenic risk score (PRS) analysis. The y-axis refers to the PRS tested (schizophrenia, bipolar disorder, major depressive disorder, attention deficit/hyperactivity disorder (ADHD), autism spectrum disorder, neuroticism and intelligence) and x-axis represents the odds ratio (OR). Points display the OR and 95% confidence intervals (error bars) for each PRS regressed against each psychotic experiences phenotype; visual hallucinations (dark blue), auditory hallucinations (light blue), delusions of reference (orange) and delusions of persecution (green).

© 2019 Legge SE et al. *JAMA Psychiatry*.

eFigure 11. PRS Analysis of Psychotic Experience Symptoms

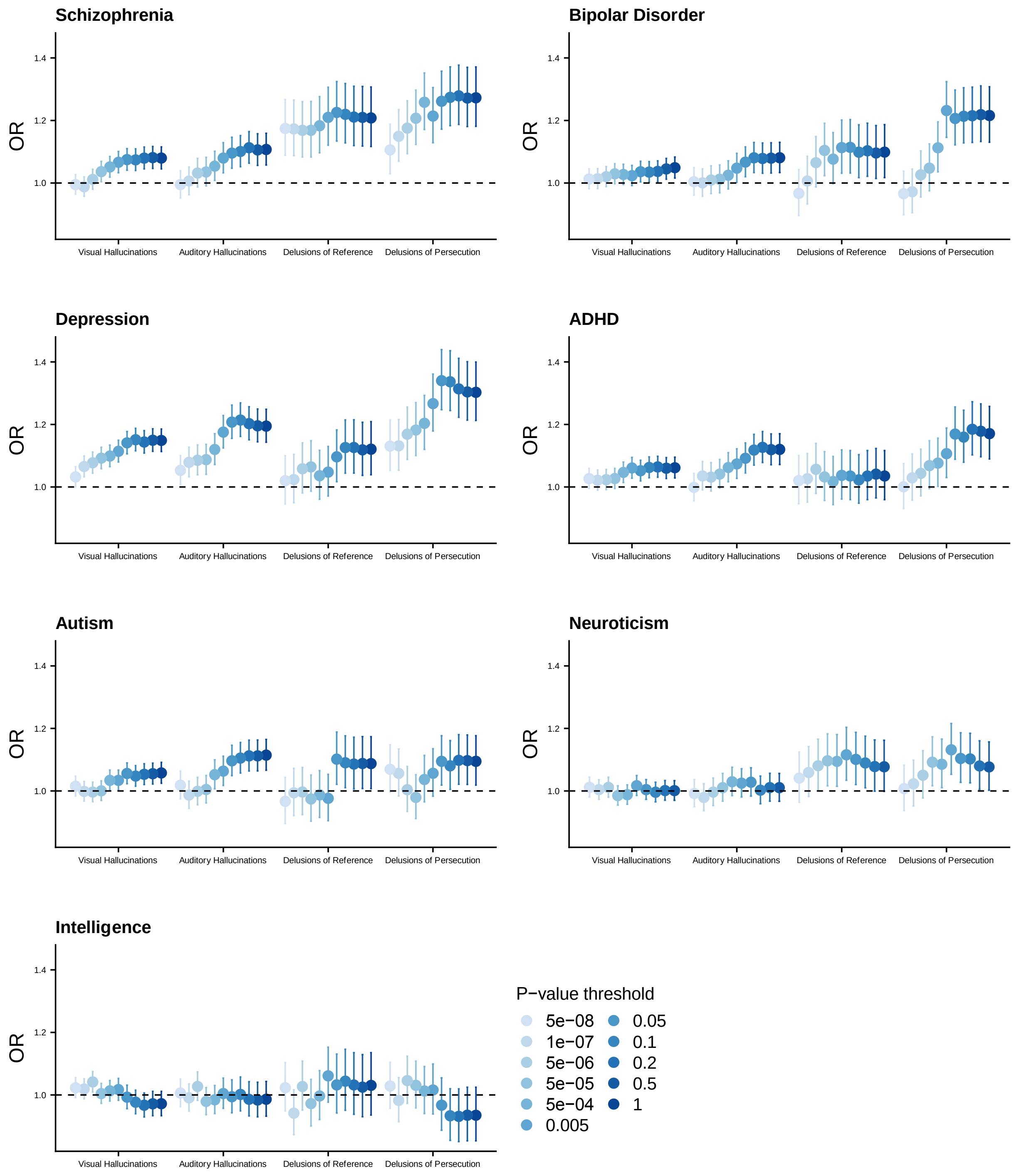

Polygenic risk score (PRS) analysis. Each plot shows results for each PRS (schizophrenia, bipolar disorder, major depressive disorder, attention deficit/hyperactivity disorder (ADHD), autism spectrum disorder, neuroticism and intelligence). The x-axis shows the psychotic experience phenotype (visual hallucinations, auditory hallucinations, delusions of reference, delusions of persecution). Points display the odds ratio (OR) and error bars represent the 95% confidence intervals for the OR.

© 2019 Legge SE et al. *JAMA Psychiatry*.

eFigure 12. PRS Analysis of PE Symptoms

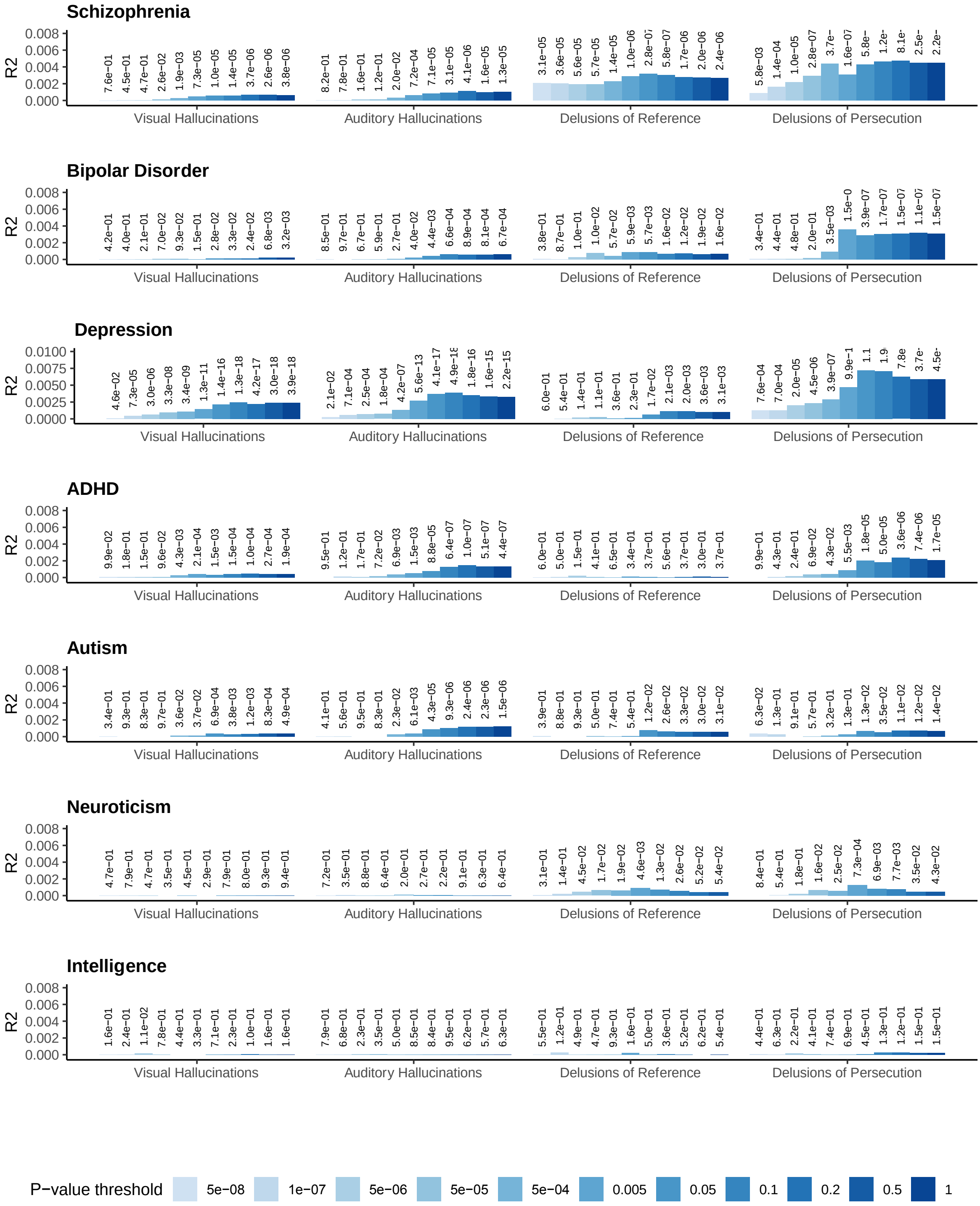

Polygenic risk score (PRS) analysis. Each plot shows results for each PRS (schizophrenia, bipolar disorder, major depressive disorder, attention deficit/hyperactivity disorder (ADHD), autism spectrum disorder, neuroticism and intelligence). The x-axis shows the psychotic experience phenotype (visual hallucinations, auditory hallucinations, delusions of reference, delusions of persecution). Bars represent adjusted variance explained (R^2) and association p-values are listed above.

© 2019 Legge SE et al. *JAMA Psychiatry*.

eTable 1. Validation of Psychotic Experience GWAS in ALSPAC Cohort

Age (years)	Psychotic experience (PE) measure	PRS p-value threshold	Effect†	SE	p-value	lower 95% CI	upper 95% CI	pseudo r2	N
12 and/or 18	Definite versus suspected PE or none	0.5	1.126	0.057	0.020	1.019	1.245	0.0019	5310
12 and/or 18	Definite versus suspected PE or none	0.4	1.126	0.057	0.020	1.019	1.244	0.0019	5310
12 and/or 18	Definite versus suspected PE or none	0.3	1.126	0.057	0.020	1.019	1.244	0.0019	5310
12 and/or 18	Definite versus suspected PE or none	0.2	1.137	0.058	0.012	1.028	1.256	0.0022	5310
12 and/or 18	Definite versus suspected PE or none	0.1	1.110	0.057	0.041	1.004	1.227	0.0014	5310
12 and/or 18	Definite versus suspected PE or none	0.05	1.113	0.057	0.036	1.007	1.230	0.0015	5310
12 and/or 18	Definite versus suspected PE or none	0.01	1.090	0.056	0.090	0.987	1.205	0.0010	5310
12 and/or 18	Definite versus suspected PE or none	0.001	1.099	0.056	0.065	0.994	1.214	0.0012	5310
12 and/or 18	Definite versus suspected PE or none	0.0001	1.009	0.052	0.869	0.912	1.116	0.0000	5310
12 and/or 18	Definite versus suspected PE or none	0.00001	1.030	0.054	0.566	0.931	1.141	0.0001	5310
13 and/or 18	Definite versus suspected PE or none	0.000001	0.924	0.047	0.123	0.837	1.022	0.0008	5310
14 and/or 18	Definite versus suspected PE or none	0.0000001	0.903	0.045	0.039	0.819	0.995	0.0014	5310
12 and/or 18	Definite versus suspected PE or none	0.00000005	0.947	0.051	0.308	0.852	1.052	0.0004	5310
18	Suspected or definite PE versus none	0.5	1.163	0.070	0.012	1.034	1.308	0.0031	3407
18	Suspected or definite PE versus none	0.4	1.165	0.070	0.011	1.035	1.311	0.0032	3407
18	Suspected or definite PE versus none	0.3	1.164	0.070	0.011	1.035	1.310	0.0032	3407
18	Suspected or definite PE versus none	0.2	1.184	0.072	0.005	1.052	1.333	0.0039	3407
18	Suspected or definite PE versus none	0.1	1.197	0.073	0.003	1.063	1.348	0.0044	3407
18	Suspected or definite PE versus none	0.05	1.254	0.076	0.000	1.113	1.412	0.0070	3407
18	Suspected or definite PE versus none	0.01	1.210	0.073	0.002	1.075	1.362	0.0050	3407
18	Suspected or definite PE versus none	0.001	1.233	0.075	0.001	1.094	1.390	0.0059	3407
18	Suspected or definite PE versus none	0.0001	1.062	0.065	0.327	0.941	1.198	0.0005	3407
18	Suspected or definite PE versus none	0.00001	1.113	0.070	0.088	0.984	1.260	0.0015	3407
18	Suspected or definite PE versus none	0.000001	0.954	0.059	0.447	0.846	1.077	0.0003	3407
18	Suspected or definite PE versus none	0.0000001	0.945	0.056	0.343	0.841	1.062	0.0004	3407
18	Suspected or definite PE versus none	0.00000005	0.991	0.060	0.886	0.880	1.117	0.0000	3407
18	Definite versus suspected PE or none	0.5	1.203	0.096	0.020	1.030	1.406	0.0042	3407
18	Definite versus suspected PE or none	0.4	1.200	0.096	0.022	1.027	1.403	0.0040	3407
18	Definite versus suspected PE or none	0.3	1.192	0.095	0.027	1.020	1.393	0.0037	3407
18	Definite versus suspected PE or none	0.2	1.232	0.099	0.009	1.053	1.441	0.0052	3407
18	Definite versus suspected PE or none	0.1	1.216	0.098	0.015	1.039	1.424	0.0046	3407

© 2019 Legge SE et al. *JAMA Psychiatry*.

18	Definite versus suspected PE or none	0.05	1.247	0.100	0.006	1.066	1.458	0.0059	3407
18	Definite versus suspected PE or none	0.01	1.217	0.097	0.014	1.041	1.422	0.0047	3407
18	Definite versus suspected PE or none	0.001	1.200	0.096	0.023	1.025	1.404	0.0040	3407
18	Definite versus suspected PE or none	0.0001	0.962	0.078	0.633	0.821	1.128	0.0002	3407
18	Definite versus suspected PE or none	0.00001	0.991	0.080	0.910	0.845	1.161	0.0000	3407
18	Definite versus suspected PE or none	0.000001	0.852	0.066	0.039	0.732	0.992	0.0031	3407
18	Definite versus suspected PE or none	0.0000001	0.871	0.065	0.067	0.752	1.010	0.0024	3407
18	Definite versus suspected PE or none	0.00000005	0.880	0.079	0.156	0.738	1.050	0.0017	3407
16.5	PE latent trait	0.5	0.021	0.028	0.460	-0.034	0.076	-	3604
16.5	PE latent trait	0.4	0.028	0.028	0.320	-0.027	0.083	-	3604
16.5	PE latent trait	0.3	0.023	0.028	0.422	-0.032	0.078	-	3604
16.5	PE latent trait	0.2	0.005	0.028	0.858	-0.050	0.060	-	3604
16.5	PE latent trait	0.1	0.012	0.028	0.664	-0.043	0.067	-	3604
16.5	PE latent trait	0.05	0.032	0.028	0.256	-0.023	0.087	-	3604
16.5	PE latent trait	0.01	0.026	0.028	0.357	-0.029	0.081	-	3604
16.5	PE latent trait	0.001	0.015	0.028	0.598	-0.040	0.070	-	3604
16.5	PE latent trait	0.0001	0.050	0.027	0.067	-0.003	0.103	-	3604
16.5	PE latent trait	0.00001	0.020	0.028	0.479	-0.035	0.075	-	3604
16.5	PE latent trait	0.000001	0.045	0.029	0.117	-0.012	0.102	-	3604
16.5	PE latent trait	0.0000001	0.058	0.028	0.039	0.003	0.113	-	3604
16.5	PE latent trait	0.00000005	0.058	0.028	0.038	0.003	0.113	-	3604
18	Any anxiety disorder	0.5	1.049	0.060	0.404	0.938	1.174	0.0003	3296
18	Any anxiety disorder	0.4	1.040	0.060	0.492	0.930	1.164	0.0002	3296
18	Any anxiety disorder	0.3	1.040	0.060	0.492	0.930	1.164	0.0002	3296
18	Any anxiety disorder	0.2	1.032	0.059	0.588	0.922	1.155	0.0001	3296
18	Any anxiety disorder	0.1	1.053	0.061	0.373	0.940	1.179	0.0004	3296
18	Any anxiety disorder	0.05	1.081	0.062	0.173	0.966	1.210	0.0009	3296
18	Any anxiety disorder	0.01	1.008	0.057	0.886	0.902	1.127	0.0000	3296
18	Any anxiety disorder	0.001	1.065	0.061	0.272	0.952	1.192	0.0006	3296
18	Any anxiety disorder	0.0001	1.017	0.059	0.769	0.907	1.141	0.0000	3296
18	Any anxiety disorder	0.00001	1.040	0.061	0.498	0.928	1.166	0.0002	3296
18	Any anxiety disorder	0.000001	0.978	0.057	0.708	0.872	1.097	0.0001	3296
18	Any anxiety disorder	0.0000001	1.043	0.061	0.470	0.930	1.171	0.0002	3296
18	Any anxiety disorder	0.00000005	1.048	0.059	0.401	0.939	1.170	0.0003	3296
18	ICD-10 depression	0.5	1.187	0.077	0.009	1.044	1.348	0.0039	3296

© 2019 Legge SE et al. *JAMA Psychiatry*.

18	ICD-10 depression	0.4	1.176	0.077	0.013	1.035	1.337	0.0035	3296
18	ICD-10 depression	0.3	1.168	0.076	0.017	1.028	1.327	0.0032	3296
18	ICD-10 depression	0.2	1.140	0.075	0.045	1.003	1.296	0.0022	3296
18	ICD-10 depression	0.1	1.146	0.075	0.038	1.008	1.303	0.0024	3296
18	ICD-10 depression	0.05	1.093	0.071	0.172	0.962	1.241	0.0010	3296
18	ICD-10 depression	0.01	1.072	0.069	0.285	0.944	1.216	0.0006	3296
18	ICD-10 depression	0.001	1.216	0.079	0.003	1.070	1.382	0.0051	3296
18	ICD-10 depression	0.0001	1.067	0.071	0.325	0.937	1.215	0.0005	3296
18	ICD-10 depression	0.00001	1.035	0.068	0.600	0.910	1.178	0.0002	3296
18	ICD-10 depression	0.000001	1.052	0.071	0.457	0.921	1.201	0.0003	3296
18	ICD-10 depression	0.0000001	1.072	0.072	0.300	0.940	1.223	0.0006	3296
18	ICD-10 depression	0.00000005	1.027	0.066	0.673	0.906	1.165	0.0001	3296

Validation of psychotic experiences GWAS in ALSPAC cohort. Columns represent age at which psychotic experience measure is based, the psychotic experience measure targeted, the PRS training p-value threshold for SNP inclusion, effect size (see below notes), standard error (SE), p-value, lower 95% confidence interval of the effect size, upper 95% confidence interval of effect size, pseudo r*2* and total number of individuals included (N).

† Note that for PEs measured at 12 and 18 years, effect sizes represent odds ratios per standard deviation increase in PRS. For the PE latent trait at age 16.5 years effect sizes represent standard deviation change in latent trait per standard deviation change in PRS.

© 2019 Legge SE et al. *JAMA Psychiatry*.

eTable 2. Genetic Correlations

**Trait 1**	**Trait 2**	**r_g**	**SE**	**P-value**
Any Psychotic Experience	ADHD	0.2392	0.0844	4.61E-03
Any Psychotic Experience	Autism	0.3853	0.1024	1.68E-04
Any Psychotic Experience	Intelligence	0.11	0.0802	1.70E-01
Any Psychotic Experience	Major Depressive Disorder	0.4617	0.0701	4.64E-11
Any Psychotic Experience	Bipolar Disorder	0.1495	0.0685	2.92E-02
Any Psychotic Experience	Schizophrenia	0.2087	0.0526	7.29E-05
ADHD	Autism	0.3459	0.0511	1.33E-11
ADHD	Intelligence	-0.3868	0.0441	1.74E-18
ADHD	Major Depressive Disorder	0.4266	0.0341	5.26E-36
ADHD	Bipolar Disorder	0.1164	0.0409	4.49E-03
ADHD	Schizophrenia	0.1679	0.033	3.62E-07
Autism	Intelligence	0.2679	0.0464	7.79E-09
Autism	Major Depressive Disorder	0.4379	0.0391	4.30E-29
Autism	Bipolar Disorder	0.1321	0.0491	7.10E-03
Autism	Schizophrenia	0.2379	0.0411	7.02E-09
Intelligence	Major Depressive Disorder	-0.0238	0.0339	4.82E-01
Intelligence	Bipolar Disorder	0.0031	0.0385	9.36E-01
Intelligence	Schizophrenia	-0.1493	0.0305	9.71E-07
Major Depressive Disorder	Bipolar Disorder	0.3435	0.0283	7.97E-34
Major Depressive Disorder	Schizophrenia	0.3389	0.0226	1.05E-50
Bipolar Disorder	Schizophrenia	0.6668	0.0212	3.42E-216
Neuroticism	Any Psychotic Experience	0.3996	0.184	2.99E-02
Neuroticism	ADHD	0.3415	0.1336	1.06E-02
Neuroticism	Autism	0.215	0.1427	1.32E-01
Neuroticism	Intelligence	-0.1938	0.1095	7.68E-02
Neuroticism	Major Depressive Disorder	0.8006	0.121	3.65E-11
Neuroticism	Bipolar Disorder	0.1943	0.1058	6.65E-02
Neuroticism	Schizophrenia	0.2176	0.075	3.72E-03

Genetic correlation analysis. Columns represent the traits (Trait 1 and Trait 2) that the correlations were calculated for, the genetic correlation (r_g), standard error (SE) and p-value.

© 2019 Legge SE et al. *JAMA Psychiatry*.

eTable 3. Polygenic Risk Score Analysis

Schizophrenia PRS						
**Phenotype**	**Cases**	**Controls**	**R^2**	**AUC**	**OR (95% CI)**	**P**
Any PE vs. controls	6,132	122,066	0.0011	0.512	1.09 (1.06-1.12)	2.96E-11
Distressing PE vs. controls	2,146	122,066	0.0026	0.531	1.18 (1.13-1.23)	4.86E-13
Distressing PE vs. non-distressing PE	2,146	3,810	0.0042	0.514	1.13 (1.07-1.19)	2.30E-05
Non-distressing PE vs. controls	3,804	122,066	0.0002	0.503	1.05 (0.01-1.08)	8.25E-03
Multiple PEs vs. controls	3,343	122,066	0.0013	0.515	1 .11 (1.08-1.15)	2.13E-09
Visual hallucinations	4,053	123,985	0.0006	0.507	1.08 (1.04-1.11)	1.04E-05
Auditory hallucinations	2,026	126,831	0.0008	0.511	1.10 (1.05-1.15)	7.05E-05
Delusions of reference	677	128,401	0.0032	0.526	1.23 (1.13-1.32)	2.75E-07
Delusions of persecution	755	128,414	0.0043	0.554	1.26 (1.17-1.36)	5.84E-10
Bipolar Disorder PRS						
**Phenotype**	**Cases**	**Controls**	**R^2**	**AUC**	**OR (95% CI)**	**P**
Any PE vs. controls	6,132	122,066	0.0006	0.507	1.07 (1.04-1.10)	5.11E-07
Distressing PE vs. controls	2,146	122,066	0.002	0.524	1.15 (1.10-1.20)	3.77E-10
Distressing PE vs. non-distressing PE	2,146	3,810	0.0036	0.514	1.11 (1.05-1.17)	9.17E-05
Non-distressing PE vs. controls	3,804	122,066	0.0001	0.501	1.03 (1.00-1.06)	7.90E-02
Multiple PE vs. controls	3,343	122,066	0.0007	0.51	1.08 (1.04-1.12)	1.53E-05
Visual hallucinations	4,053	123,985	0.0001	0.501	1.04 (1.00-1.07)	0.0279
Auditory hallucinations	2,026	126,831	0.0004	0.507	1.07 (1.02-1.12)	4.39E-03
Delusions of reference	677	128,401	0.0009	0.505	1.11 (1.03-1.20)	5.70E-03
Delusions of persecution	755	128,414	0.0029	0.539	1.21 (1.12-1.30)	3.90E-07
Major depressive disorder PRS						
**Phenotype**	**Cases**	**Controls**	**R^2**	**AUC**	**OR (95% CI)**	**P**
Any PE vs. controls	6,132	122,066	0.0032	0.525	1.16 (1.13-1.19)	1.48E-30
Distressing PE vs. controls	2,146	122,066	0.0049	0.542	1.24 (1.19-1.29)	5.07E-23
Distressing PE vs. non-distressing PE	2,146	3,810	0.0036	0.511	1.11 (1.05-1.17)	8.24E-05
Non-distressing PE vs. controls	3,804	122,066	0.0015	0.516	1.12 (1.08-1.15)	3.87E-11
Multiple PE vs. controls	3,343	122,066	0.0038	0.531	1.19 (1.15-1.24)	5.21E-24
Visual hallucinations	4,053	123,985	0.0022	0.52	1.14 (1.11-1.18)	1.44E-16
Auditory hallucinations	2,026	126,831	0.0037	0.533	1.21 (1.16-1.26)	4.13E-17
Delusions of reference	677	128,401	0.0001	0.506	1.10 (1.02-1.18)	0.017
Delusions of persecution	755	128,414	0.0072	0.571	1.34 (1.25-1.44)	1.11E-15
ADHD PRS						
**Phenotype**	**Cases**	**Controls**	**R^2**	**AUC**	**OR (95% CI)**	**P**
Any PE vs. controls	6,132	122,066	0.0005	0.505	1.06 (1.03-1.09)	5.73E-06
Distressing PE vs. controls	2,146	122,066	0.0003	0.506	1.06 (1.01-1.10)	0.012
Distressing PE vs. non-distressing PE	2,146	3,810	0.00002	0.500	0.99 (0.94-1.05)	0.794
Non-distressing PE vs. controls	3,804	122,066	0.0005	0.505	1.06 (1.03-1.10)	2.09E-04
Multiple PE vs. controls	3,343	122,066	0.0008	0.511	1.09 (1.05-1.13)	1.23E-06
Visual hallucinations	4,053	123,985	0.0003	0.504	1.05 (1.02-1.09)	1.51E-03
Auditory hallucinations	2,026	126,831	0.0008	0.507	1.09 (1.04-1.14)	8.83E-05

© 2019 Legge SE et al. *JAMA Psychiatry*.

Delusions of reference	677	128,401	0.0001	0.500	1.04 (0.96-1.12)	0.370
Delusions of persecution	755	128,414	0.0021	0.533	1.17 (1.09-1.26)	1.84E-05
Autism spectrum disorder PRS						
**Phenotype**	**Cases**	**Controls**	**R^2**	**AUC**	**OR (95% CI)**	**P**
Any PE vs. controls	6,132	122,066	0.0007	0.507	1.07 (1.04-1.10)	1.34E-07
Distressing PE vs. controls	2,146	122,066	0.0009	0.517	1.10 (1.05-1.15)	1.70E-05
Distressing PE vs. non-distressing PE	2,146	3,810	0.0004	0.502	1.04 (0.98-1.09)	0.167
Non-distressing PE vs. controls	3,804	122,066	0.0004	0.504	1.06 (1.02-1.09)	5.90E-04
Multiple PE vs. controls	3,343	122,066	0.0010	0.511	1.10 (1.06-1.13)	2.51E-07
Visual hallucinations	4,053	123,985	0.0004	0.504	1.06 (1.02-1.09)	6.94E-04
Auditory hallucinations	2,026	126,831	0.0009	0.510	1.10 (1.05-1.15)	4.31E-05
Delusions of reference	677	128,401	0.0008	0.505	1.10 (1.02-1.19)	0.012
Delusions of persecution	755	128,414	0.0007	0.516	1.10 (1.02-1.18)	0.013
Neuroticism PRS						
**Phenotype**	**Cases**	**Controls**	**R^2**	**AUC**	**OR (95% CI)**	**P**
Any PE vs. controls	6,132	122,066	0.00001	0.501	1.03 (1.00-1.05)	0.043
Distressing PE vs. controls	2,146	122,066	0.00001	0.506	1.06 (1.02-1.11)	5.05E-03
Distressing PE vs. non-distressing PE	2,146	3,810	0.0012	0.505	1.06 (0.01-1.12)	0.025
Non-distressing PE vs. controls	3,804	122,066	0.000004	0.5	1.00 (0.97-1.03)	0.988
Multiple PE vs. controls	3,343	122,066	0.0003	0.502	1.05 (1.01-1.09)	6.15E-03
Visual hallucinations	4,053	123,985	0.0002	0.502	1.02 (0.99-1.05)	0.29
Auditory hallucinations	2,026	126,831	0.0001	0.501	1.02 (0.98-1.07)	0.271
Delusions of reference	677	128,401	0.0009	0.509	1.12 (1.03-1.20)	4.56E-03
Delusions of persecution	755	128,414	0.0013	0.526	1.13 (1.05-1.22)	7.31E-04
Intelligence PRS						
**Phenotype**	**Cases**	**Controls**	**R^2**	**AUC**	**OR (95% CI)**	**P**
Any PE vs. controls	6,132	122,066	-0.000002	0.5	1.01 (0.98-1.04)	0.684
Distressing PE vs. controls	2,146	122,066	-0.000002	0.5	1.00 (0.95-1.05)	0.998
Distressing PE vs. non-distressing PE	2,146	3,810	0.0001	0.501	0.98 (0.92-1.05)	0.578
Non-distressing PE vs. controls	3,804	122,066	0.00003	0.501	1.02 (0.98-1.06)	0.348
Multiple PE vs. controls	3,343	122,066	0.00004	0.5	1.02 (0.98-1.06)	0.414
Visual hallucinations	4,053	123,985	-0.00002	0.5	0.99 (0.96-1.03)	0.715
Auditory hallucinations	2,026	126,831	0.00001	0.501	0.99 (0.94-1.05)	0.836
Delusions of reference	677	128,401	0.00002	0.496	1.03 (0.94-1.13)	0.496
Delusions of persecution	755	128,414	0.00007	0.505	0.97 (0.89-1.05)	0.45

Polygenic risk scores (PRS) were calculated in UK Biobank for schizophrenia, bipolar disorder, major depressive disorder, attention deficit/hyperactivity disorder (ADHD), autism spectrum disorder, neuroticism and intelligence. Each of the PRS (at p-value training threshold of $p < 0.05$) was regressed against different psychotic experience phenotypes. Columns in each table (which corresponds to each PRS) refer to the psychotic experience phenotype assessed, the number of cases, number of controls, variance explained (R^2), area under the curve (AUC), odds ratio (OR) and 95% confidence interval, and p-value (P).

© 2019 Legge SE et al. *JAMA Psychiatry*.

eTable 4. Polygenic Risk Score Analysis—Covarying for Schizophrenia PRS

Bipolar Disorder PRS						
**Phenotype**	**Cases**	**Controls**	**R^2**	**AUC**	**OR (95% CI)**	**P**
Any PE vs. controls	6,132	122,066	0.00027	0.5019	1.05 (1.02-1.08)	9.03E-04
Distressing PE vs. controls	2,146	122,066	0.00197	0.5242	1.15 (1.10-1.20)	3.77E-10
Distressing PE vs. non-distressing PE	2,146	3,810	0.00178	0.508	1.08 (1.02-1.14)	5.38E-03
Multiple PE vs. controls	3,343	122,066	0.00028	0.502	1.05 (1.02-1.09)	5.35E-03
Visual hallucinations	4,053	123,985	0.00003	0.5001	1.02 (0.98-1.05)	0.306
Auditory hallucinations	2,026	126,831	0.00017	0.5015	1.04 (0.99-1.09)	6.81E-02
Delusions of reference	677	128,401	0.00024	0.5008	1.06 (0.98-1.15)	1.61E-01
Delusions of persecution	755	128,414	0.00137	0.509	1.14 (1.06-1.23)	4.58E-04
Major depressive disorder PRS						
**Phenotype**	**Cases**	**Controls**	**R^2**	**AUC**	**OR (95% CI)**	**P**
Any PE vs. controls	6,132	122,066	0.00282	0.5181	1.15 (1.12-1.18)	7.45E-27
Distressing PE vs. controls	2,146	122,066	0.0041	0.5226	1.22 (1.17-1.27)	1.73E-19
Distressing PE vs. non-distressing PE	2,146	3,810	0.0027	0.5067	1.10 (1.04-1.16)	6.13E-04
Multiple PE vs. controls	3,343	122,066	0.00325	0.5204	1.18 (1.14-1.22)	4.64E-21
Visual hallucinations	4,053	123,985	0.00192	0.516	1.13 (1.10-1.17)	8.39E-15
Auditory hallucinations	2,026	126,831	0.0033	0.5256	1.20 (1.15-1.25)	1.63E-15
Delusions of reference	677	128,401	0.00037	0.5038	1.07 (0.99-1.16)	8.05E-02
Delusions of persecution	755	128,414	0.00593	0.5344	1.31 (1.22-1.41)	3.33E-13

Polygenic risk score analysis (PRS) as described in **eTable3** but including the schizophrenia PRS as a covariate.

© 2019 Legge SE et al. *JAMA Psychiatry*.

eTable 5. Depression Polygenic Risk Score Analysis—Removing Individuals With Depression

Major depressive disorder PRS						
**Phenotype**	**Cases**	**Controls**	**R^2**	**AUC**	**OR (95% CI)**	**P**
Any PE vs. controls	3,635	96,546	0.00143	0.5146	1.11 (1.08-1.15)	4.92E-10
Distressing PE vs. controls	1,022	96,546	0.00249	0.5323	1.18 (1.11-1.25)	2.61E-07
Distressing PE vs. non-distressing PE	1,022	3,547	0.00216	0.5058	1.09 (1.01-1.17)	2.08E-02
Non-distressing PE vs. controls	1,882	96,546	0.00155	0.5155	1.13 (1.08-1.18)	3.18E-07
Multiple PE vs. controls	2,490	97,617	0.00118	0.5130	1.11 (1.06-1.15)	5.55E-07
Visual hallucinations	1,108	99,595	0.00138	0.5171	1.13 (1.06-1.20)	8.02E-05
Auditory hallucinations	383	100,422	0.00001	0.4986	1.03 (0.93-1.14)	5.23E-01
Delusions of reference	290	100,603	0.00438	0.5563	1.28 (1.14-1.44)	3.18E-05
Delusions of persecution	3,635	96,546	0.00143	0.5146	1.11 (1.08-1.15)	4.92E-10

Polygenic risk score analysis (PRS) for major depressive disorder as described in **eTable3** but excluding all individuals that reported a lifetime diagnosis of depression.

© 2019 Legge SE et al. *JAMA Psychiatry*.

eTable 6. Association of Neuropsychiatric Polygenic Risk Scores With Completion of the Mental Health Questionnaire

Schizophrenia PRS						
**P-value threshold**	**R2**	**AUC**	**OR**	**L95**	**U95**	**P**
0.00000005	0.00019	0.501	0.975	0.969	0.982	6.32E-14
0.0000001	0.00021	0.501	0.974	0.968	0.980	4.74E-15
0.000005	0.00032	0.501	0.968	0.961	0.974	1.91E-22
0.00005	0.00040	0.502	0.964	0.958	0.970	1.08E-27
0.0005	0.00060	0.503	0.956	0.950	0.962	1.17E-40
0.005	0.00082	0.503	0.949	0.942	0.955	1.09E-54
0.05	0.00097	0.504	0.944	0.938	0.950	1.12E-64
0.1	0.00104	0.504	0.942	0.936	0.948	3.24E-69
0.2	0.00110	0.505	0.940	0.934	0.947	1.01E-72
Bipolar Disorder PRS						
**P-value threshold**	**R2**	**AUC**	**OR**	**L95**	**U95**	**P**
0.00000005	0.00001	0.500	1.005	0.998	1.011	1.74E-01
0.0000001	0.00002	0.500	1.009	1.002	1.015	9.00E-03
0.000005	0.00003	0.500	1.009	1.003	1.016	6.42E-03
0.00005	0.00000	0.500	1.001	0.994	1.008	7.55E-01
0.0005	0.00000	0.500	1.003	0.997	1.010	3.48E-01
0.005	0.00000	0.500	1.001	0.994	1.007	8.49E-01
0.05	0.00000	0.500	1.000	0.994	1.007	9.13E-01
0.1	0.00000	0.500	1.002	0.995	1.008	6.32E-01
0.2	0.00000	0.500	1.002	0.995	1.008	6.43E-01
Major depressive disorder PRS						
**P-value threshold**	**R2**	**AUC**	**OR**	**L95**	**U95**	**P**
0.00000005	0.00001	0.500	0.994	0.987	1.001	1.16E-01
0.0000001	0.00007	0.500	0.984	0.977	0.992	2.33E-05
0.000005	0.00008	0.500	0.984	0.976	0.991	7.08E-06
0.00005	0.00018	0.501	0.976	0.969	0.983	3.15E-11
0.0005	0.00024	0.501	0.972	0.965	0.979	7.20E-15
0.005	0.00052	0.503	0.958	0.951	0.965	1.55E-30
0.05	0.00092	0.504	0.945	0.938	0.952	1.35E-52
0.1	0.00094	0.504	0.944	0.938	0.951	7.44E-54
0.2	0.00097	0.504	0.943	0.937	0.950	1.43E-55
Neuroticism PRS						
**P-value threshold**	**R2**	**AUC**	**OR**	**L95**	**U95**	**P**
0.00000005	0.00038	0.502	1.036	1.029	1.043	4.07E-26
0.0000001	0.00047	0.502	1.040	1.034	1.047	4.23E-32
0.000005	0.00071	0.503	1.050	1.043	1.057	4.58E-48
0.00005	0.00149	0.506	1.073	1.066	1.080	4.62E-98
0.0005	0.00286	0.511	1.103	1.096	1.110	8.28E-187
0.005	0.00398	0.514	1.123	1.116	1.131	1.57E-258
0.05	0.00537	0.518	1.145	1.138	1.153	1.00E-300
0.1	0.00552	0.518	1.147	1.140	1.155	1.00E-300

© 2019 Legge SE et al. *JAMA Psychiatry*.

0.2	0.00545	0.518	1.147	1.139	1.154	1.00E-300
Intelligence PRS						
**P-value threshold**	**R2**	**AUC**	**OR**	**L95**	**U95**	**P**
0.0000001	0.00000	0.500	0.997	0.990	1.003	3.45E-01
0.000005	0.00001	0.500	0.996	0.990	1.003	2.49E-01
0.00005	0.00001	0.500	1.006	0.999	1.012	9.36E-02
0.0005	0.00000	0.500	0.997	0.991	1.004	3.87E-01
0.005	0.00007	0.500	0.984	0.978	0.991	2.62E-06
0.05	0.00030	0.501	0.969	0.963	0.975	4.57E-21
0.1	0.00031	0.501	0.969	0.962	0.975	1.80E-21
0.2	0.00032	0.501	0.968	0.962	0.974	2.68E-22
0.5	0.00029	0.501	0.969	0.963	0.976	1.78E-20

Polygenic risk scores (PRS) analysis of completion of the Mental Health Questionnaire (MHQ). Each of the PRS was regressed against completion of the MHQ. Columns in each table (which corresponds to each PRS) refer to the p-value threshold for SNP inclusion of the PRS, variance explained (R^2), area under the curve (AUC), odds ratio (OR), the upper and lower 95% confidence intervals, and p-value (P).

© 2019 Legge SE et al. *JAMA Psychiatry*.

eReferences

1. Bycroft C, Freeman C, Petkova D, et al. Genome-wide genetic data on ~500,000 UK Biobank participants. *bioRxiv.* 2017.
2. Maechler M, Rousseeuw P, Croux C, et al. robustbase: Basic Robust Statistics R package version 0.93-2. 2018. http://CRAN.R-project.org/package=robustbase.
3. Hubert M, Rousseeuw PJ, Verdonck T. A Deterministic Algorithm for Robust Location and Scatter. *J Comput Graph Stat.* 2012;21(3):618-637.
4. Conomos MP, Laurie CA, Stilp AM, et al. Genetic Diversity and Association Studies in US Hispanic/Latino Populations: Applications in the Hispanic Community Health Study/Study of Latinos. *American journal of human genetics.* 2016;98(1):165-184.
5. Boyd A, Golding J, Macleod J, et al. Cohort Profile: the 'children of the 90s'--the index offspring of the Avon Longitudinal Study of Parents and Children. *International journal of epidemiology.* 2013;42(1):111-127.
6. Fraser A, Macdonald-Wallis C, Tilling K, et al. Cohort Profile: the Avon Longitudinal Study of Parents and Children: ALSPAC mothers cohort. *International journal of epidemiology.* 2013;42(1):97-110.
7. Purcell S, Neale B, Todd-Brown K, et al. PLINK: a tool set for whole-genome association and population-based linkage analyses. *American journal of human genetics.* 2007;81(3):559-575.
8. Horwood J, Salvi G, Thomas K, et al. IQ and non-clinical psychotic symptoms in 12-year-olds: results from the ALSPAC birth cohort. *Brit J Psychiat.* 2008;193(3):185-191.
9. Zammit S, Kounali D, Cannon M, et al. Psychotic Experiences and Psychotic Disorders at Age 18 in Relation to Psychotic Experiences at Age 12 in a Longitudinal Population-Based Cohort Study. *American Journal of Psychiatry.* 2013;170(7):742-750.
10. Zammit S, Hamshere M, Dwyer S, et al. A population-based study of genetic variation and psychotic experiences in adolescents. *Schizophr Bull.* 2014;40(6):1254-1262.
11. Zammit S, Owen MJ, Evans J, Heron J, Lewis G. Cannabis, COMT and psychotic experiences. *Brit J Psychiat.* 2011;199(5):380-385.
12. Jones HJ, Heron J, Hammerton G, et al. Investigating the genetic architecture of general and specific psychopathology in adolescence. *Transl Psychiat.* 2018;8.
13. Jones HJ, Stergiakouli E, Tansey KE, et al. Phenotypic Manifestation of Genetic Risk for Schizophrenia During Adolescence in the General Population. *JAMA psychiatry.* 2016;73(3):221-228.
14. Schizophrenia Working Group of the Psychiatric Genomics Consortium. Biological insights from 108 schizophrenia-associated genetic loci. *Nature.* 2014;511(7510):421-427.
15. Pardinas AF, Holmans P, Pocklington AJ, et al. Common schizophrenia alleles are enriched in mutation-intolerant genes and in regions under strong background selection. *Nature genetics.* 2018.
16. Stahl E, Forstner A, McQuillin A, et al. Genomewide association study identifies 30 loci associated with bipolar disorder. *bioRxiv.* 2017.
17. Wray NR, Ripke S, Mattheisen M, et al. Genome-wide association analyses identify 44 risk variants and refine the genetic architecture of major depression. *Nature genetics.* 2018;50(5):668-681.
18. Demontis D, Walters RK, Martin J, et al. Discovery of the first genome-wide significant risk loci for attention deficit/hyperactivity disorder. *Nature genetics.* 2019;51(1):63-75.

© 2019 Legge SE et al. *JAMA Psychiatry*.

19. Grove J, Ripke S, Als TD, et al. Identification of common genetic risk variants for autism spectrum disorder. *Nature genetics.* 2019;51(3):431-444.
20. Genetics of Personality Consortium, de Moor MH, van den Berg SM, et al. Meta-analysis of Genome-wide Association Studies for Neuroticism, and the Polygenic Association With Major Depressive Disorder. *JAMA psychiatry.* 2015;72(7):642-650.
21. Savage JE, Jansen PR, Stringer S, et al. Genome-wide association meta-analysis in 269,867 individuals identifies new genetic and functional links to intelligence. *Nature genetics.* 2018;50(7):912-919.
22. Euesden J, Lewis CM, O'Reilly PF. PRSice: Polygenic Risk Score software. *Bioinformatics.* 2015;31(9):1466-1468.
23. Kendall KM, Rees E, Escott-Price V, et al. Cognitive Performance Among Carriers of Pathogenic Copy Number Variants: Analysis of 152,000 UK Biobank Subjects. *Biological psychiatry.* 2017;82(2):103-110.
24. Wang K, Li M, Hadley D, et al. PennCNV: an integrated hidden Markov model designed for high-resolution copy number variation detection in whole-genome SNP genotyping data. *Genome research.* 2007;17(11):1665-1674.
25. Rees E, Kendall K, Pardinas AF, et al. Analysis of Intellectual Disability Copy Number Variants for Association With Schizophrenia. *JAMA psychiatry.* 2016;73(9):963-969.
26. Coe BP, Witherspoon K, Rosenfeld JA, et al. Refining analyses of copy number variation identifies specific genes associated with developmental delay. *Nature genetics.* 2014;46(10):1063-1071.
27. Pruim RJ, Welch RP, Sanna S, et al. LocusZoom: regional visualization of genome-wide association scan results. *Bioinformatics.* 2010;26(18):2336-2337.

© 2019 Legge SE et al. *JAMA Psychiatry*.
